# Transcriptome sequencing and ITRAQ reveal the detoxification mechanism of *Bacillus* GJ1, a potential biocontrol agent for Huanglongbing

**DOI:** 10.1371/journal.pone.0200427

**Published:** 2018-08-09

**Authors:** Jizhou TANG, Yuanxi DING, Jing Nan, Xiangyu YANG, Liang SUN, Xiuyun ZHAO, Ling JIANG

**Affiliations:** 1 College of Horticulture and Forestry, Key Laboratory of Horticultural Plant Biology of Ministry of Education, Huazhong Agricultural University, Wuhan, Hubei, China; 2 College of life Science and Technology, Huazhong Agricultural University, Wuhan, Hubei, China; 3 State Key Laboratory of Agricultural Microbiology, Huazhong Agricultural University, Wuhan, Hubei, China; 4 National Indoor Conservation Center of Virus-free Germplasm of Fruit Crops, Huazhong Agricultural University, Wuhan, Hubei, China; Clemson University, UNITED STATES

## Abstract

Huanglongbing (HLB) is the most serious disease affecting citrus production worldwide. No HLB-resistant citrus varieties exist. The HLB pathogen *Candidatus* Liberibacter asiaticus is nonculturable, increasing the difficulty of preventing and curing the disease. We successfully screened the biocontrol agent *Bacillus* GJ1 for the control of HLB in nursery-grown citrus plants. RNA sequencing (RNA-seq) of the transcriptome and isobaric tags for relative and absolute quantification of the proteome revealed differences in the detoxification responses of *Bacillus* GJ1-treated and -untreated *Ca*. L. asiaticus-infected citrus. Phylogenetic tree alignment showed that GJ1 was classified as *B*. *amyloliquefaciens*. The effect of eliminating the HLB pathogen was measured using real-time quantitative polymerase chain reaction (qPCR) and PCR. The results indicate that the rate of detoxification reached 50% after seven irrigations, of plants with an OD_600nm_≈1 *Bacillus* GJ1 suspension. Most importantly, photosynthesis-antenna proteins, photosynthesis, plant-pathogen interactions, and protein processing in the endoplasmic reticulum were significantly upregulated (padj < 0.05), as shown by the KEGG enrichment analysis of the transcriptomes; nine of the upregulated genes were validated by qPCR. Transcription factor analysis of the transcriptomes was performed, and 10 TFs were validated by qPCR. Cyanoamino acid metabolism, regulation of autophagy, isoflavonoid biosynthesis, starch and sucrose metabolism, protein export, porphyrin and chlorophyll metabolism, and carotenoid biosynthesis were investigated by KEGG enrichment analysis of the proteome, and significant differences were found in the expression of the genes involved in those pathways. Correlation analysis of the proteome and transcriptome showed common entries for the significantly different expression of proteins and the significantly different expression of genes in the GO and KEGG pathways, respectively. The above results reveal important information about the detoxification pathways.

## Introduction

Citrus is an important fruit crop worldwide. However, Huanglongbing (HLB) currently poses a serious threat to citrus production in approximately 50 countries. The HLB pathogen comprises three species of α-Proteobacterium Liberibacter, namely, “*Candidatus* Liberibacter asiaticus,” “*Candidatus* Liberibacter africanus,” and “*Candidatus* Liberibacter americanus” [1]. The pathogen acts as a parasite, primarily in the citrus phloem. *Candidatus* L. asiaticus is nonculturable and causes shoot yellowing, mottled leaves, tree debility, and death. All commercial citrus varieties lack resistance to *Ca*. L. asiaticus. Thus, effective methods for controlling HLB are urgently required in citrus production. Effective prevention and cure of HLB are challenging because an understanding of its pathogenicity is insufficient [2, 3]. Several techniques, including graft-based chemotherapy [4, 5], transgenic technology [6, 7], screening of small-molecule inhibitors [8–10], and screening to identify a tolerant combination of stock and scion [11], have been tested for controlling HLB. However, these methods have limitations and require further improvement.

*Candidatus* L. asiaticus is a nutrient-deficient bacterium that consumes host nutrients, resulting in an imbalance of the host metabolism [12]. Genome sequence analysis of citrus indicates that *Ca*. L. asiaticus encodes the bacterial flagellum component Flg22, a well-known pathogen-associated molecular pattern (PAMP) that activates plant defense mechanisms [13]. The complete genome sequence of *Ca*. L. asiaticus was obtained through metagenomics. Annotation revealed a generally high percentage of genes involved in cell motility (4.5%) and active transport (8.0%), and these genes may contribute to the virulence of *Ca*. L. asiaticus. This species exhibits limited aerobic respiration and lacks type III and IV secretion systems, in addition to lacking the extracellular degradative enzymes typically associated with free-living bacteria [12]. Infection with *Ca*. L. asiaticus alters carbohydrate metabolism in *Citrus sinensis*. The starch levels in *C*Las-infected leaves with and without symptoms are increased by 3.1- and 7.9-fold, respectively, compared with the starch levels of healthy controls [14]. Additionally, expression profiling of the genes encoding the proteins involved in starch breakdown suggests that DPE2 and MEX1 are downregulated. Freitas and colleagues investigated the role of invertases in the establishment of plant defense responses and suggested that a complex regulation of sugar signaling occurs during plant–pathogen interaction [15]. Sucrose and invertases are part of the plant defense response to biotic stresses [16]. Plants that are susceptible (navel orange) and tolerant (*Citrus volkameriana*) to HLB were analyzed by proteomics; the results show that greater amino acid degradation occurs in infected navel orange plants than in infected *C*. *volkameriana* and that four glutathione-S-transferases are upregulated in *C*. *volkameriana* but not in the navel orange. These proteins are involved in radical ion detoxification. Thus, upregulation of the proteins involved in radical ion detoxification should be considered an important mechanism for increased tolerance to HLB [17]. Study of the transcriptome, proteomics, and metabonomics of citrus can increase our understanding of the pathogenicity and detoxification mechanisms of HLB [18–22].

The highly variable prophage region is thought to have led to population differentiation of pathogenic Asian species during evolution. In addition to *Ca*. L. asiaticus, numerous endophytic bacteria are found in citrus tissues and organs, and these organisms form the microecosystem of citrus. Microbacillus, Bacillus, Pseudomonas, and Kocuria are the dominant flora in HLB-infected plants that are infected with bacteria [23]. *Bacillus* may be a commensal bacterium in citrus. Therefore, we sought to find an antagonistic bacterium that can be used to sustainably eliminate the activity of the study pathogens and restore metabolic balance in citrus.

Bacillus (*Bacillus* sp.) is a gram-positive bacterium that can form spores under aerobic or facultative anaerobic conditions. Bacillus acts as a parasite in the leaves and roots of plants during the growth process by competing with pathogens for nutrients and infection sites, secreting antimicrobial substances, inhibiting pathogen growth, inducing the host to produce systemic resistance, and resisting invasion by pathogenic bacteria to achieve biological control. During growth, Bacillus produces a series of metabolites that inhibit fungal and bacterial activities. Thus, Bacillus species have a strong inhibitory effect on plant pathogens [24]. Numerous studies have demonstrated the role of *Bacillus amyloliquefaciens* in combating plant diseases such as tomato bacterial wilt [25, 26], Fusarium wilt in banana [27], *Erwinia carotovora* in vegetables [28], *Phoma tracheiphila* in *Citrus aurantium* seedlings [29], and late blight pathogen in tomato [30] and scab in potato[31]. Cao et al. (2013) [32] identified upregulated proteins that are potentially involved in the antagonistic effects of *B*. *amyloliquefaciens* G1. *Bacillus amyloliquefaciens* subsp. plantarum GR53 resists *Rhizoctonia* disease in Chinese cabbage through hormonal and antioxidant regulation [33]. Greenhouse studies have revealed that the application of a corn flour formulation of *Bacillus subtilis* var. *amyloliquefaciens* (FZB24) as a foliar spray led to a significant (49.7%) reduction in the severity of late blight [34].

To screen biocontrol agents for HLB, we irrigated the roots of infected citrus plants with the selected strain Bacillus GJ1 and detected the pathogen in the leaves by qPCR and PCR. Bacillus GJ1 was identified as the effective strain for biocontrol in potted citrus seedling. For the comparison of the GJ1 treated and untreated plants, the transcriptome and the ITRAQ proteome of the leaves were used in this study to elucidate differences in the detoxification mechanism.

## Materials and methods

### Plant material, treatment and statistical analyses

**Fig 1:**
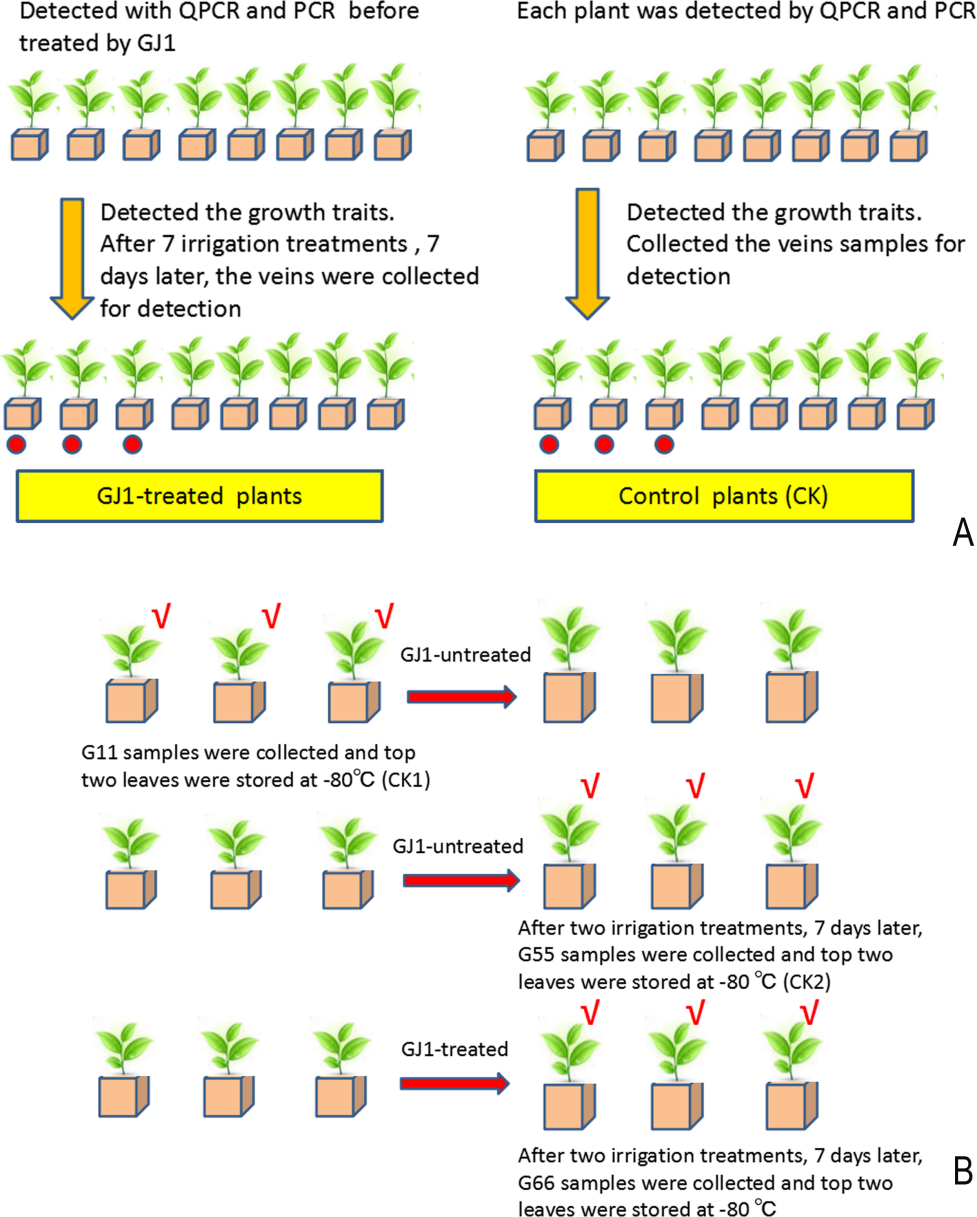
The diagram of experiment design. A: GJ1 treatment processing and sampling procedures. Red circle: labeled plants are used for proteomic analysis. B: The experiment design for transcriptome analysis. √: the top two leaves were collected to keep the sampling site consistent.

*Citrus sinensis* (L.) Osbeck plants were grown in a net house, and the plants used for testing were identified as pathogen-carrying plants by qPCR and PCR. *Citrus tangerine* Tanaka was the rootstock. The plants were grown in identical culture medium in plastic pots containing 1/3 peat mold, 1/3 vermiculite, and 1/3 normal soil; the medium was supplemented with 1/8 MS mineral element (0.5 L/plant) and 200 mg/L polypeptide (poly-γ-glutamic acid) once a month. *Bacillus* GJ1 treatment was applied via root irrigation once every 7 days; each plant was irrigated with 1.5 L of treatment solution (OD_600nm_≈1). Eight GJ1-treated and eight untreated plants were used for the HLB biocontrol test after seven irrigation treatments, we collected the samples for analysis of qPCR and PCR, The untreated plants served as the control plants for the entire experiment. The experimental design is shown in the following diagram (Fig 1). Leaf veins from leaves on different parts of the tree were collected and mixed before being stored at -80°C. One part of the vein sample was used for the qPCR detection of *Ca*. L. asiaticus, and another was used for the PCR detection of *Ca*. L. asiaticus, with three replicates per plant. Among the 16 plants, three GJ1-treated and three untreated plants were used for proteome analysis of ITRAQ and RT-PCR validation tests separately; the leaves for these tests were collected on day 7 after the final treatment of seven irrigation treatments. Eight GJ1-treated and eight untreated plants were used for observations on the growth traits (plant height, crown diameter, number of new shoots, trunk diameter, and root cap diameter) after the seven irrigation treatments. Statistical analyses of the growth traits were performed with “the hypothesis test for the comparison of two samples averages.” The subsequent statistical analyses of the *Ca*. Liberibacter asiaticus pathogen-carrying condition were performed with “the continuous correction of the hypothesis test for the comparison of two sample percentages,” as shown in S1 Table.

The other nine plants were used for transcriptome analyses, with the growth condition and management methods being the same as those described above. *Bacillus* GJ1 was applied via root irrigation once every 20 days, and GJ1-treated and untreated leaves were collected after two irrigation treatments and again after 7 days after the final treatment (these samplings were called G66 and G55, respectively); another control sample, G11, was collected 27 days earlier. Each group of samples had three biological replicates.

### Bacterial strain isolation and determination

The bacterial strains were isolated from healthy leaves of *Citrus sinensis* (L.) Osbeck “Newhall.” The leaves were conventionally sterilized and washed with sterile water; the leaves were then ground in PBS (pH 7) buffer, and the ground material was used to inoculate the LB medium. Three bacterial strains were isolated; trial testing showed that strain GJ1 was effective against HLB. The bacterial strains were preserved in nutrient broth as glycerol stocks at -80°C for further use.

### Amplification and alignment of 16S rDNA sequence

Genomic DNA of Bacillus GJ1 was extracted using a Pure Plasmid Mini Kit (Comwin Biotech Co., Ltd., Beijing, China). A nucleic acid and protein analyzer (Amersham, Biochrom Ltd. Cambridge CB4 OFJ, England) and a 17R microcentrifuge (Thermo Fisher Scientific) were used. The 16S rDNA region of the ribosomal DNA was amplified using primers 16S (F): 5'-agagtttgatcctggctcag-3' and 16S (R): 5'-ggttaccttgttacgactt-3'. The *rec*A gene was amplified using the primers *rec*A(+): 5'-tgagtgatcgtcaggcagccttag-3' and *rec*A(-): 5'-ttcttcataagaataccacgaaccgc-3' [34]. The PCR conditions were 94°C for 4 min, followed by 35 cycles of 95°C for 1 min, 55°C for 1 min, and 72°C for 1 min. The amplified fragment was inserted into a pGEM-T vector, and Sangon Biotech (Shanghai) sequenced the PCR products. Multiple alignments were performed using DNAman software.

### qPCR and PCR detection of HLB after treatment with *Bacillus* GJ1

Total DNA was extracted by the CTAB method. qPCR was performed using SYBR Green qPCR Master Mix (TaKaRa) according to the manufacturer’s instructions. The primer sequences used in the real-time qPCR assay are shown in S1 Table. Sangon Shanghai Biology Technology, Ltd., synthesized the primers. qPCR was performed using a Rotor Gene 6000 (Corbett). The cycling conditions began with 2 min of polymerase activity at 95°C, followed by 40 cycles of 95°C for 20 s, 58–60°C for 20 s, and 72°C for 20 s. Each assay was conducted in triplicate, and a no-template control was included. The relative expression level was analyzed using the 2^−ΔΔCt^ method [35]. Only primer sets producing a single sequence-specific peak in the dissociation curve were used. The data were analyzed using the Rotor Gene 6000 Series software (VIRTUAL Mode software package). The data are presented as the mean ± standard error of three replicates. The housekeeping gene used in this study was the cytochrome oxidase gene; primer sequences of primers (COX+, COX-) are shown in Table A in S1 Table, and the fragment length was 68 bp [36]. The target gene amplification was the specific sequence for the Asian citrus HLB pathogen ribosomal protein gene rplJ/rplL; the amplification fragment length was 87 bp, and the primers (A04 +, A04 -) are shown in Table A in S1 Table [37]. Healthy plant material was used for the negative control, and plasmid DNA that contained the ribosomal protein gene sequence was used for the positive control. Total DNA was extracted by the CTAB method, and PCR amplification was performed in a Bio-Rad amplification instrument. The primers (P400+, P400-) and (OI1, OI2) were used for amplification; the cycling conditions of the PCR were 94°C for 5 min, followed by 33 cycles of 95°C for 5 min, 56°C for 30 s, and 72°C for 90 s. The primers are shown in Table A in S1 Table.

### Validation and comparison of selected genes using log_2_(FC) and qPCR

*Citrus sinensis* was used for the qPCR validation. Total RNA was extracted from citrus leaves using RNAiso Plus (TaKaRa), and cDNA was obtained using a PrimeScript™ RT Reagent Kit with gDNA Eraser (Perfect Real Time). qPCR amplification was performed with SYBR Green Real-time PCR Master Mix (TaKaRa), and three replicates were amplified. The primers used in the qPCR amplification are listed in Table A in S1 Table. The actin gene was chosen as the constitutively expressed internal control for normalization; the other operations were the similar to those for the Huanglongbing detection procedure.

### Library preparation for transcriptome sequencing

Three group samples—G11, G55, and G66—were collected and prepared for transcriptomic analysis, and each group contained three biological replicates. RNA was extracted, and the purity was checked with the NanoPhotometer® spectrophotometer (IMPLEN, CA, USA). Sequencing libraries were generated using a NEBNext® Ultra™ RNA Library Prep Kit for Illumina® (NEB, USA), according to the manufacturer’s recommendations, and index codes were added to attribute each sequence to the appropriate sample. Briefly, mRNA was purified, and RNA fragmentation was performed in the presence of divalent cations at elevated temperature in NEBNext First Strand Synthesis Reaction Buffer (5X). The library fragments were purified using the AMPure XP system (Beckman Coulter, Beverly, MA, USA). PCR was performed with Phusion High-Fidelity DNA Polymerase, universal PCR primers, and Index (X) Primer. Finally, the PCR products were purified using the AMPure XP system, and the library quality was assessed using the Agilent Bioanalyzer 2100 system.

### Quantification of gene expression levels and differential expression analysis

Gene model annotation files for *C*. *sinensis* were directly downloaded from the genome website at http://citrus.hzau.edu.cn/orange/download/csi.chromosome.fa.tar.gz. HTSeq v0.6.1 was used to count the read numbers mapped to each gene. The FPKM of each gene was then calculated based on the length of the gene and the read count mapped to the gene [38]. Differential expression analysis of the two groups was performed using the DESeq R package (1.18.0). DESeq provides statistical routines for determining differential expression in digital gene expression data using a model based on a negative binomial distribution. The resulting P values were adjusted; genes for which P < 0.05 according to DESeq were considered differentially expressed.

### GO and KEGG enrichment analysis of differentially expressed genes

GO enrichment analysis of differentially expressed genes was implemented using the goseq R package. GO terms with corrected P values < 0.05 were considered significantly enriched among the differentially expressed genes. KEGG (http://www.genome.jp/kegg/) is a database resource for understanding high-level functions and large-scale molecular data sets generated by genome sequencing. We used KOBAS software to test for significant enrichment of differentially expressed genes in KEGG pathways and to predict gene functions (http://citrus.hzau.edu.cn/orange/download/csi.peptide.fa.tar.gz).

### Protein preparation and ITRAQ labeling

Samples were milled to powder and mixed with 1 mL of lysis buffer containing Tris base pH = 8, 7 M urea, 2 M thiourea, 0.1% SDS, 2 mM EDTA, protease inhibitor cocktail (Roche), and 1 mM phenylmethylsulfonyl fluoride in a glass homogenizer. The homogenates were incubated and then centrifuged. The supernatant was transferred to a new tube. Protein concentrations were determined using the Bradford assay. The supernatant obtained from each sample was digested with trypsin (Promega, Madison, WI, USA) at 37°C for 16 h. The peptides obtained by trypsin digestion were dried by vacuum centrifugation. Desalted peptides were labeled with iTRAQ reagents (ITRAQ® Reagent-8PLEX Multiplex Kit, 4381663) according to the manufacturer's instructions (AB Sciex, Foster City, CA). The labeled peptides were incubated for 2 h at room temperature. Differentially labeled peptides were mixed. The abovementioned treatment and control samples had three biological replicates.

### HPLC fractionation and LC-MS/MS analysis

The first dimension RP separation by MicroLC was performed on an L-3000 HPLC System (Rigol) using a Durashell RP column. Mobile phases A (2% acetonitrile, 20 mM NH_4_FA, and pH adjusted to 10.0 using NH_3_•H_2_O) and B (98% acetonitrile, 20 mM NH_4_FA, and pH adjusted to 10.0 using NH_3_•H_2_O) were used to develop a gradient elution. Fractions from the first dimension RPLC were dissolved in loading buffer and separated on a C18 column (75 μm inner diameter, 360 μm outer diameter × 10 cm, 3 μm C18). Mobile phase A consisted of 0.1% formic acid in water, and mobile phase B consisted of 0.1% formic acid in acetonitrile; a series of linear gradients, adjusted according to the hydrophobicity of the fractions eluted in 1D LC, were applied at a flow rate of 300 nL/min. For Orbitrap Q-Exactive, the source was operated at 1.8 kV. For full MS survey scanning, the AGC target was 3e6, and the scan range was from 350 to 1800, with a resolution of 70,000. The 20 most intense peaks with charge state 2 and above were selected for fragmentation by HCD with normalized collision energy of 32% for the ITRAQ-labeled peptide. The MS2 spectra were acquired at a resolution of 17,500.

### Data analysis for protein identification

The MS raw data files from Q Exactive were first searched by Mascot (Matrix Science, London, UK; version 2.6.0). Mascot searched the files using a fragment ion mass tolerance of 0.020 Da and a parent ion tolerance of 10.0 PPM. Protein identifications were accepted when they could be established at greater than 90.0% probability to achieve an FDR less than 10.0% and contained at least two identified peptides. Only ratios with P values ≤ 0.05 were used, and only ratios > 1.2 were considered significant.

### GO and KEGG enrichment analysis of differentially expressed proteins

We used two databases to predict gene functions: GO (Gene Ontology, http://www.geneontology.org) and KEGG (Kyoto Encyclopedia of Genes and Genomes, http://www.genome.jp/kegg/). GO is an internationally standardized gene function classification system and provides a dynamically updated controlled vocabulary that describes genes and gene product attributes in the organism. KEGG is a collection of manually drawn pathway maps that represent the current knowledge of molecular interactions and reaction networks.

## Results

### Identification of *Bacillus* GJ1

**Fig 2:**
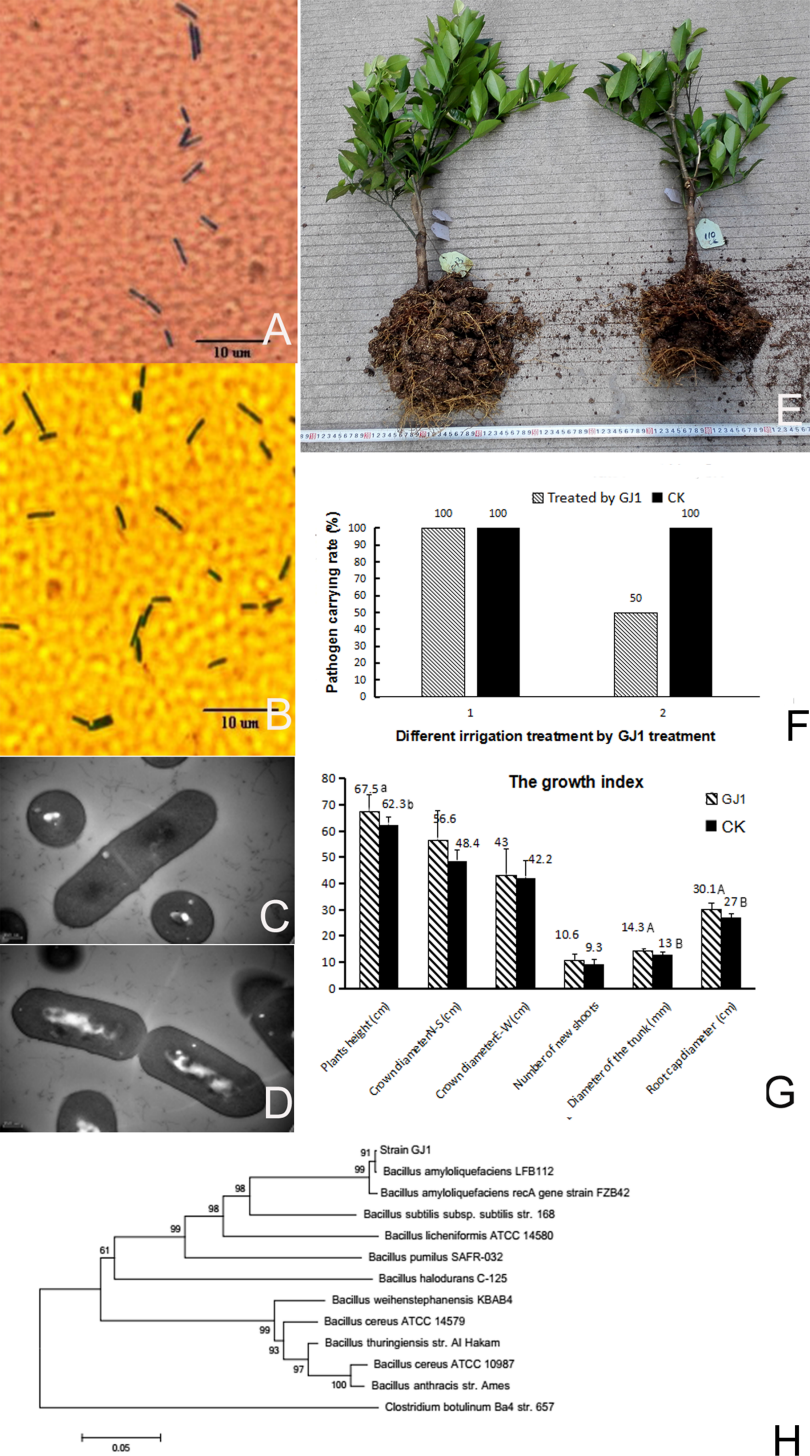
**(A-H) Identification of *Bacillus* GJ1, detection of HLB, and determination of the growth index in infected plants treated with GJ1**. *Bacillus* GJ1 was inoculated into citrus plants and observed after Gram staining (ocular: 10×, objective lens: 40×). (B) After colonization of the citrus leaves, *Bacillus* GJ1 was isolated and Gram-stained (ocular: 10×, objective lens: 40×). (C and D) TEM images of cell division of *Bacillus* GJ1 (bar: 200 nm). (E) Comparison of citrus growth conditions (left: treated with GJ1, right: CK). (F) Detection of HLB with qPCR and PCR after *Bacillus* GJ1 treatment: 1, before GJ1 treatment; 2, after seven irrigation treatments. (G) Comparison of the citrus growth traits. (H) Phylogenetic tree alignment. Two different lowercase letters indicate a significant difference between the two averages or percentages. Two different capital letters indicate a very significant difference between the two averages or percentages. The absence of letters indicates no significant difference between the two averages.

The inoculated *Bacillus* GJ1 strains isolated from the leaves appeared purple when observed under an optical microscope, and the bacteria were inferred to be gram-positive. *Bacillus* GJ1 displayed a short rod-shaped morphology, and the morphological characteristics of the inoculated and colonized bacilli in the leaves were similar. This result indicated that *Bacillus* GJ1 successfully colonized the citrus leaves (Fig 2A and 2B). The cells were approximately 1.4–1.5 μm in length, and their widths were 0.5–0.7 μm, as revealed by TEM (Fig 2C and 2D). The superstructure of Bacillus GJ1 was observed during cell division. An appropriate protocol for the preparation of TEM samples for *Bacillus* GJ1 was determined, and the morphological changes that occurred during cell division were observed. At the beginning of cell division, a diaphragm formed within the cell, followed by the formation of an integrated septum and wall; cytokinesis was then complete (Fig 2C and 2D).

### Detection of HLB by qPCR and PCR after Bacillus GJ1 treatment

*Bacillus* GJ1 was tested for its action against *Ca*. L. asiaticus in potted HLB-infected citrus plants. The roots maintained a healthy and dynamic condition after the GJ1 treatment, whereas some brown roots were observed in the control plants (Fig 2E). The presence of *Candidatus* L. asiaticus was determined strictly using qPCR and PCR. A standard curve was constructed according to the copy number of the plasmid DNA (pMD18T-*rplJ*) and corresponding CT value (Figures A-C in S1 Fig); the amplification efficiency was 99.5%. According to the copy number of citrus plant DNA and the corresponding CT value, the amplification efficiency of *COX* was 105.2% (Figures D-F in S1 Fig). When a positive result was detected either by qPCR or by PCR with the primers (p400+, P400-) and by PCR with the primers (pOI1, pOI2), the entire plant was considered infected. after seven irrigation treatments, 50% of the plants became negative for *Ca*. L. asiaticus (Fig 2F). By contrast, untreated plants remained positive for *Ca*. L. asiaticus (Tables B-D in S1 Table). However, the detoxification rate after seven irrigation treatments with *Bacillus* GJ1 was not significantly different according to “the continuous correction of the hypothesis test for the comparison of two samples percentages” t-test (| t | < t_0.05_ = 2.145), GJ1 treatment reduced or eliminated effectively the pathogen of HLB in some organ.

The roots of the control plants were partially brown at the beginning of the experiment, and the plants showed obvious changes after treatment with GJ1. The growth conditions of the citrus plants are shown in Fig 2E. Several key growth traits were investigated after the GJ1 treatment. Plant height, trunk diameter, and root cap diameter were larger than those aspects of the control plants (Fig 2G, Tables E-K in S1 Table). The differences in plant height, trunk diameter, and root cap diameter reached significant, very significant, and very significant levels, respectively, according to the hypothesis test for the comparison of two sample averages. Plant height, crown diameter (N-S), diameter (E-W), number of new shoots, trunk diameter, and root cap diameter in the treated plants changed by 8.3%, 16.9%, -1.9%, 13.9%, 10%, and 11.5%, respectively.

### Identification of Bacillus GJ1 and classification with phylogenetic tree analysis

A 1439-bp fragment was obtained by 16S rDNA sequencing. The sequence of this fragment was compared with the sequences reported in the GenBank database by BLAST and aligned using DNAman. The results indicated that the GJ1 16S rDNA sequence shared 100% homology with those of the 29 other *Bacillus* strains (Figure G in S1 Fig); of these 29 strains, 12 belonged to *B*. *amyloliquefaciens*, 12 to *B*. *subtilis*, five to *Bacillus velezensis*, and one to *Bacillus* sp. Phylogenetic tree alignment of the *recA* gene sequence showed that GJ1 was classified into *B*. *amyloliquefaciens* by multiple sequence alignments with *B*. *amyloliquefaciens* LFB112 (CP006952), *B*. *amyloliquefaciens* FZB42 (CP000560.1), *B*. *cereus* ATCC 14579 (NC_004722.1), *B*. *weihenstephanensis* KBAB4 (NC_010184.1), *B*. *cereus* ATCC 10987 (NC_003909.8), *B*. *thuringiensis* str. Al Hakam (NC_008600.1), *B*. *licheniformis* ATCC 14580 (NC_006270.3), *Clostridium botulinum* Ba4 str. 657 (NC_012658.1), *B*. *pumilus* SAFR-032 (CP000813.4), *B*. *halodurans* C-125 (BA000004.3), and *B*. *subtilis* subsp. subtilis str. 168 (NC_000964.3) (Fig 2H).

### Alignment conditions and read distribution between reads and reference genome

A total of 56.5 million and 56.7 million raw reads were generated from the control samples of the G11 and G55 groups, respectively. A total of 53.7 million raw reads were generated from G66. We obtained an average of 8.2, 8.2, and 7.7 Gbp with average GC contents of 44.0%, 43.8%, and 44.1% in G11, G55, and G66, respectively. An average of 89.4% of the 53.5 million average total reads was mapped (S2 Table). Total reads ranging from 47.4 million to 64.6 million were obtained from each sample, and 88.4–90.3% of the total reads were directly mapped to the *C*. *sinensis* genome (http://citrus.hzau.edu.cn/orange/); 84.1–85.8% of the uniquely mapped reads matched annotated citrus genes (S2 Table). Furthermore, the genetic distribution of the reads showed that 93.5–96.7% of the mapped reads corresponded to exons, whereas the remaining reads were distributed among introns (1.3–1.8%) and intergenic regions (2.8–4.7%) (S2 Fig). These results indicated that our RNA-seq results provided an appropriate data set for further exploration of the citrus transcriptome.

### Histogram of GO classification and analysis

To obtain a broad classification of the entire set of DEGs, GO enrichment analysis was conducted. All GO-enriched DEGs identified in the citrus gene database were downregulated in response to the GJ1 treatment in the G66 group compared with those treatments in the G55 group (padj < 0.05). A total of 13,224 downregulated genes were enriched. When the G66 group was compared to the G11 group, 16,448 downregulated and 272 upregulated genes were enriched (S3 Table).

**Fig 3:**
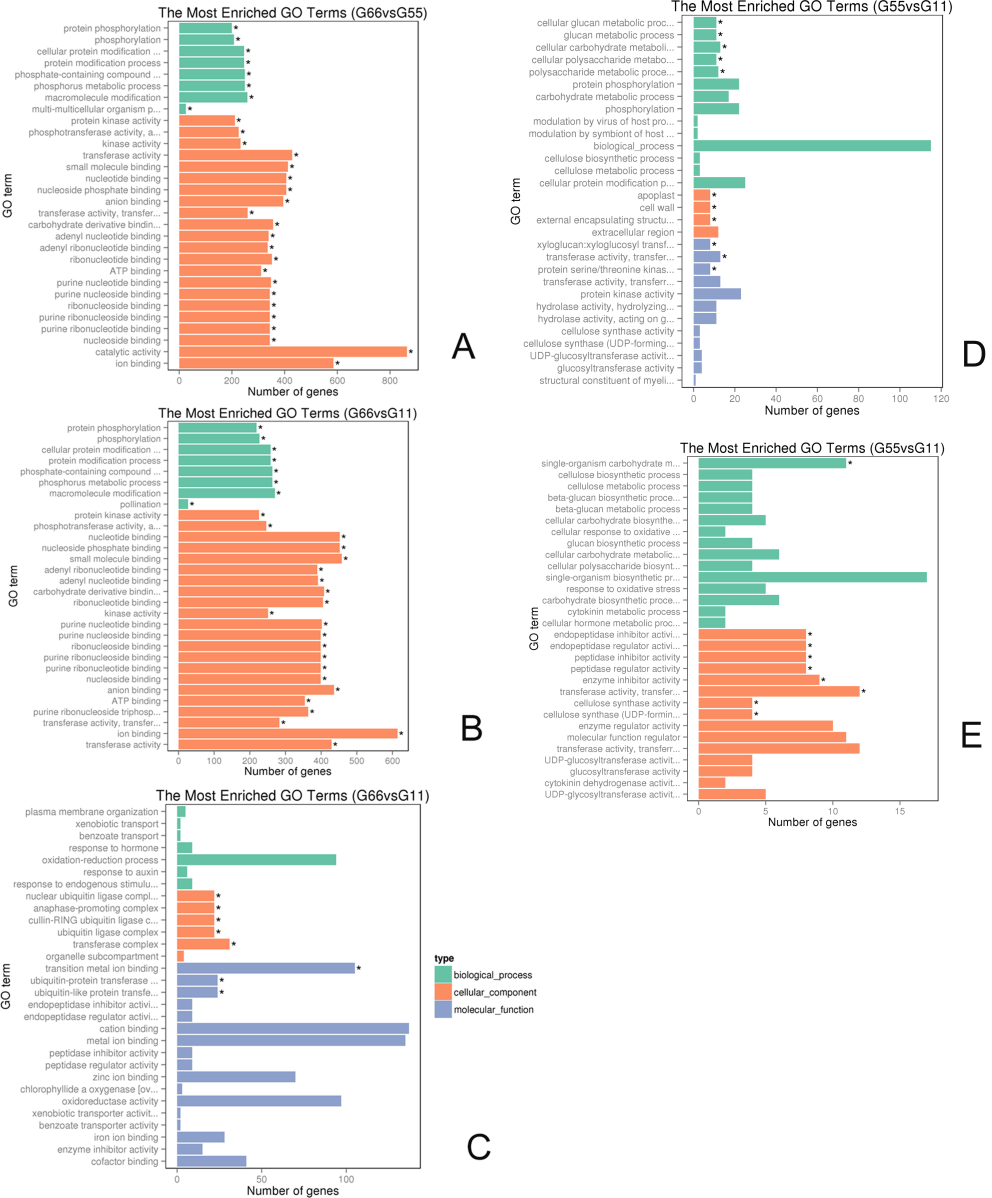
**(A-E) Analysis of the differential expression of GO-enriched genes in *Bacillus* GJ1-treated and untreated citrus plants.** (A) G66 versus G55, downregulated genes. (B) G66 versus G11, downregulated genes. (C) G66 versus G11, upregulated genes. (D) G55 versus G11, downregulated genes. (E) G55 versus G11, upregulated genes. The graph shows the GO term on the axis and the number of differentially expressed genes at the end of the bar for each GO term.

Differences in GO gene enrichment were found in the three main categories, namely, the biological process, cellular component, and molecular function. For the G66 versus the G55 experiment, we selected the 30 most significant GO terms; these terms are shown in Fig 3A. The significantly enriched biological processes included 22 processes (padj < 0.05); the top three processes were protein phosphorylation, phosphorylation, and protein modification. Twenty-eight molecular functions were significantly enriched (padj < 0.05), with protein kinase activity, transferase activity, and kinase activity being the top three. Significantly enriched cellular components included extracellular matrix and proteinaceous extracellular matrix (S3 Table and Fig 3A).

GO enrichment analysis of the differentially expressed genes allowed the identification of 12 gene types in the “biological process” that were significantly downregulated after *Bacillus* GJ1 treatment. The top three processes associated with these gene types were protein phosphorylation, phosphorylation, and the cellular protein modification process. Only three gene types for “cellular component” (apoplast, extracellular matrix, and proteinaceous extracellular matrix) were downregulated (S3 Table and Fig 3B). A total of 32 gene types were identified under the “molecular function”; the top three functions were protein kinase activity, phosphotransferase activity, and nucleotide binding, and these types were significantly downregulated after *Bacillus* GJ1 treatment in the G66 versus G11 groups. Additionally, significantly enriched upregulated cellular components included a nuclear ubiquitin ligase complex, anaphase-promoting complex, cullin-RING ubiquitin ligase complex, ubiquitin ligase complex, and transferase complex. The significantly enriched upregulated molecular functions included transition metal ion binding and ubiquitin-protein transferase activity (padj < 0.05) (S3 Table and Fig 3C).

GO enrichment analysis of the differentially expressed genes identified five gene types for the “biological process,” which were significantly downregulated in G55 compared to G11. The top three processes associated with these gene types were the cellular glucan metabolic process, glucan metabolic process, and cellular carbohydrate metabolic process. Only three gene types of “cellular component” (apoplast, cell wall, and external encapsulating structure) were significantly downregulated. Three gene types for “molecular function” were significantly downregulated, with the top three functions xyloglucan:xyloglucosyl transferase activity, transferase activity, and protein serine/threonine kinase activity (S3 Table and Fig 3D). Additionally, the significantly enriched upregulated “biological process” only included a single-organism carbohydrate metabolic process, whereas eight gene types for the “molecular function” were significantly upregulated, with the top three functions being endopeptidase inhibitor activity, endopeptidase regulator activity, and peptidase inhibitor activity (padj < 0.05) (S3 Table and Fig 3E). From the above information, a difference in gene expression over time is illustrated.

### Analysis of differentially expressed genes using KEGG enrichment scatter plots

**Fig 4:**
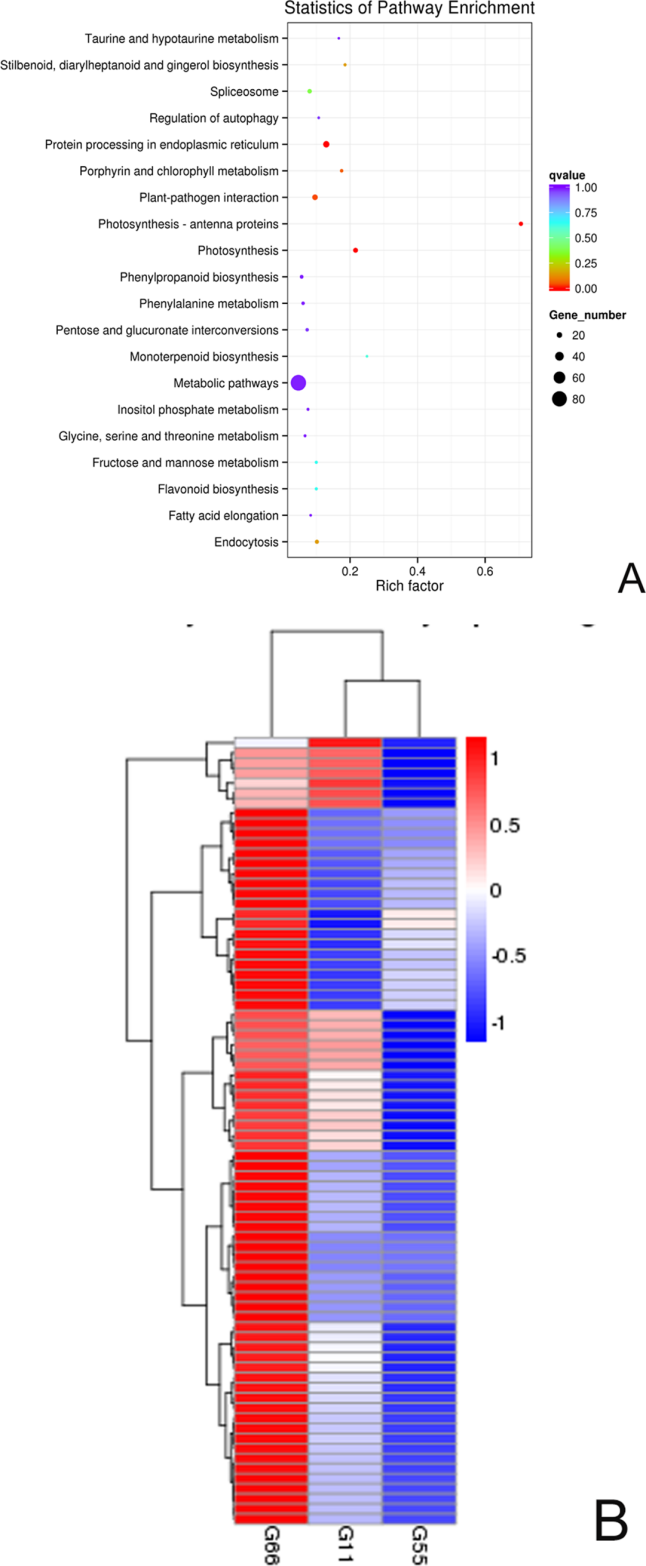
**(A-B) Scatter plot and heat map analysis of differentially expressed genes in the *Bacillus* GJ1 test**. (A) Scatter plot analysis of regulated genes in the transcriptomes of G66 and G55. (B) Hierarchical clustering map of differentially expressed genes in the control groups (G11 and G55) and the *Bacillus* GJ1-treated group (G66). The genes in the top five enriched pathways are shown as red points in the scatter plot. Red indicates highly expressed genes, and blue indicates genes with low expression.

We selected 20 of the most abundant pathway entries displayed in the scatter plot graph shown in Fig 4A. The scale of the input number/background number was 241/8432 in the upregulated DEG-enriched KEGG pathway in the G66 versus G55 experiment. The four DEG-enriched KEGG pathways included photosynthesis-antenna proteins, photosynthesis, protein processing in the endoplasmic reticulum, and plant-pathogen interactions (padj < 0.05); other upregulated pathways included porphyrin and chlorophyll metabolism; stilbenoid, diarylheptanoid, and gingerol biosynthesis; endocytosis, spliceosome, and monoterpenoid biosynthesis; fructose and mannose metabolism; flavonoid biosynthesis; regulation of autophagy, pentose and glucuronate interconversions; inositol phosphate metabolism, phenylalanine metabolism, taurine and hypotaurine metabolism; phenylpropanoid biosynthesis; glycine, serine and threonine metabolism; and fatty acid elongation. In the G55 versus G11 experiment, only one DEG-enriched KEGG pathway, zeatin biosynthesis, was significantly upregulated (padj < 0.05); no DEG-enriched KEGG pathway was significantly downregulated (S4 Table and S5 Table).

### Clustering analysis of differentially expressed genes

Clustering analysis was performed to determine the pattern of differential gene expression under various experimental conditions. We conducted DEG KEGG pathway-enrichment analysis (padj < 0.05) of the top upregulated metabolic pathways that were regulated by GJ1 treatment, as shown in Fig 4B and S4–S6 Tables. To further select the most significant pathways, a total of 9374 genes were mapped across 241 genes in different metabolic pathways in the G66 versus G55 groups. Pathways with at least 73 differentially upregulated genes were short-listed in four pathways (S4 Table). A total of 59 differentially downregulated genes were grouped in the two pathways—plant-pathogen interaction and alpha-linolenic acid metabolism (padj < 0.05) (S5 Table). To further select the most significant pathways in the G66 versus G11 groups, the pathways with at least 64 differentially downregulated genes in the two pathways—the plant–pathway interaction and alpha-linolenic acid metabolism (padj < 0.05)—were short-listed (S6 Table).

Hierarchical clustering analysis of the differentially expressed genes was performed based on the pairwise comparisons shown in Fig 4B. The four top metabolic pathways that were upregulated by the *Bacillus* GJ1 treatment of the plants in the G66 group are shown in red in Fig 4B. The genes shown in blue are for the G55 control group. With the exception of the genes Cs1g05950, Cs1g06360, Cs1g11320, Cs1g11390, Cs1g12050, Cs1g12920 and Cs1g12930, which were upregulated, the log_2_ (FPKM+1) values of the other genes in the G11 control group were higher than those in the G55 group. All other genes shown in blue in the G11 control group were obviously downregulated compared with those in the G66 *Bacillus* GJ1 treatment group (S7 Table). The functions of these genes in the four enriched and upregulated pathways (padj < 0.05) are described below.

#### Improvement in the ability to capture light

In the category of photosynthesis-antenna proteins, the upregulated unigenes were associated with the light-harvesting chlorophyll protein complex (LHC), namely, Lhca1 (Cs7g27290), Lhca2 (Cs7g10740), and Lhca3 (Cs3g13920). These genes, which encode proteins that form part of photosystem I, were upregulated 1.87-, 1.11-, and 1.48-fold, respectively. Lhcb1 (Cs2g03780), Lhcb2 (Cs2g19680), Lhcb3 (Cs1g06360), Lhcb4 (Cs6g08260), Lhcb5 (Cs5g18620), and Lhcb6 (orange1.1t03504) encode proteins that form part of photosystem II of photosynthesis (Fig 4B and S3 Fig). These genes were upregulated 2.21-, 1.48-, 1.68-, 2.04-, 1.39-, and 1.88-fold, respectively.

#### Efficient absorption of light energy

In photosynthesis, the upregulated unigenes PsbQ (Cs2g07330) and PsbY (Cs7g09900) encode proteins that belong to photosystem II. PsbQ is the most divergent of the extrinsic proteins of PSII in higher plants. These unigenes were upregulated 1.40- and 1.42-fold, respectively. PsaD (Cs5g31180), PsaE (Cs5g34450), PsaK (Cs2g09520), PsaL (Cs2g18170), and PsaO (Cs6g12390), which belong to photosystem I (Fig 4B and S4 Fig), were upregulated 1.45-, 1.67-, 1.03-, 1.32-, and 1.81-fold, respectively.

#### Recognition of a misfolded protein, enforcement of chaperones, and folding catalysts

During protein processing in the endoplasmic reticulum, the upregulated unigenes that encode the heat shock proteins Hsp70 (Cs8g18620), Hsp90 (Cs9g19220 and Cs5g03150), and sHSF (Cs8g19520, Cs8g19530, Cs7g10040, Cs1g16780, and Cs8g19540) were upregulated 1.86-, 2.89-, 3.76-, 3.14-, 2.59-, 2.28-, 2.40-, and 2.08-fold, respectively (Fig 4B and S5 Fig). The products of these unigenes could act as chaperones and folding catalysts and participate in endoplasmic reticulum-associated degradation (ERAD), which directs the degradation of misfolded or inappropriate proteins.

#### RPS2- and HSPs-induced defense responses

During plant-pathogen interaction, the upregulated expression of CDPK, CMCML, and MEKK1 induces the expression of defense-related genes. The bacterial secretion system stimulates RPS2 upregulation and further induces HSP90-upregulated expression, leading to strengthening of the hypersensitive response (HR). The RPS2 Cs4g13040 (1.8243), Novel00091 (1.2968), and 13 RPS2 genes were upregulated; HSP(90) Cs5g03150 and Cs9g19220 were upregulated by 3.76- and 2.88-fold, respectively (Fig 4B and S6 Fig).

In addition, in porphyrin and chlorophyll metabolism, the upregulated (padj = 0.058) unigenes participate in metabolism of cofactors and vitamins. Cs9g13460 play a role in the transformation of protoporphyin IX into magnesium protoporphyrin IX, whereas Cs7g19710 plays a part in the transformation of magnesium protoporphyrin IX into magnesium protoporphyrin IX 13-monomethyl ester. Three Cs6g16200 genes catalyze and trend the aerobic pathway, whereas two BchP genes (Cs5g10740) participate in bacterio-pheophytins and drive chlorophyllide a to change into bacteriochlorophyll a or bacteriochlorophyll b. These genes were upregulated 1.24, 1.24, 1.53, and 1.7 times, respectively. Cs3g19690 (upregulated 1.12 times.) impels chlorophyllide a to change into chlorophyll a, ultimately resulting in pheophytins (Fig 4B; S7 Fig).

### Validation of RNA-seq results by qPCR

**Fig 5:**
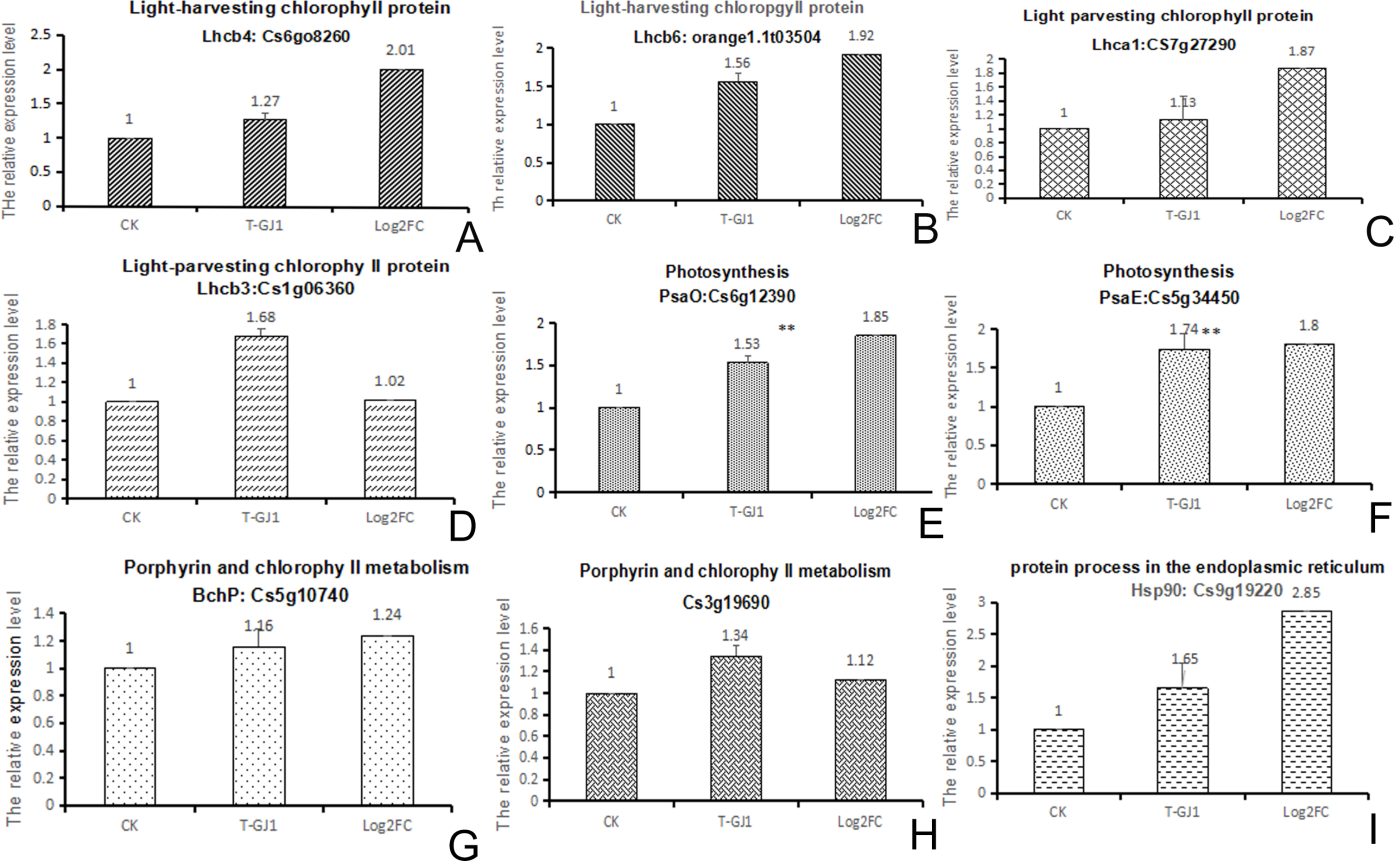
**(A-I) Comparison of the RNA-seq and qPCR results validate the upregulation of gene expression**. The graph shows the fold change in expression of genes in *Bacillus* GJ1-treated samples relative to the control and relative expression by qPCR. * indicates P < 0.05 and ** indicates P < 0.01, based on t-tests.

Based on the KEGG enrichment pathways, four pathways related to photosynthesis were upregulated (padj < 0.05). Among the observed pathways, the expression of nine genes was validated by qPCR. Comparison of the log_2_(FC) values showed that all nine of these genes, namely, Cs6g08260, orange1.1t03504, Cs7g07290, Cs1g06360, Cs12390, Cs5g34450, Cs7g10740, Cs3g19690, and Cs9g19220, showed similar upregulated expression in the results obtained by RNA-seq and qPCR (Fig 5). Although the samples were collected from different batches, these results indicated a consistent trend. The specificity of the qPCR products was verified by melting curve analysis (S8 Fig).

### Transcription factor analysis by RNA-seq and partial validation by qRT-PCR

**Fig 6:**
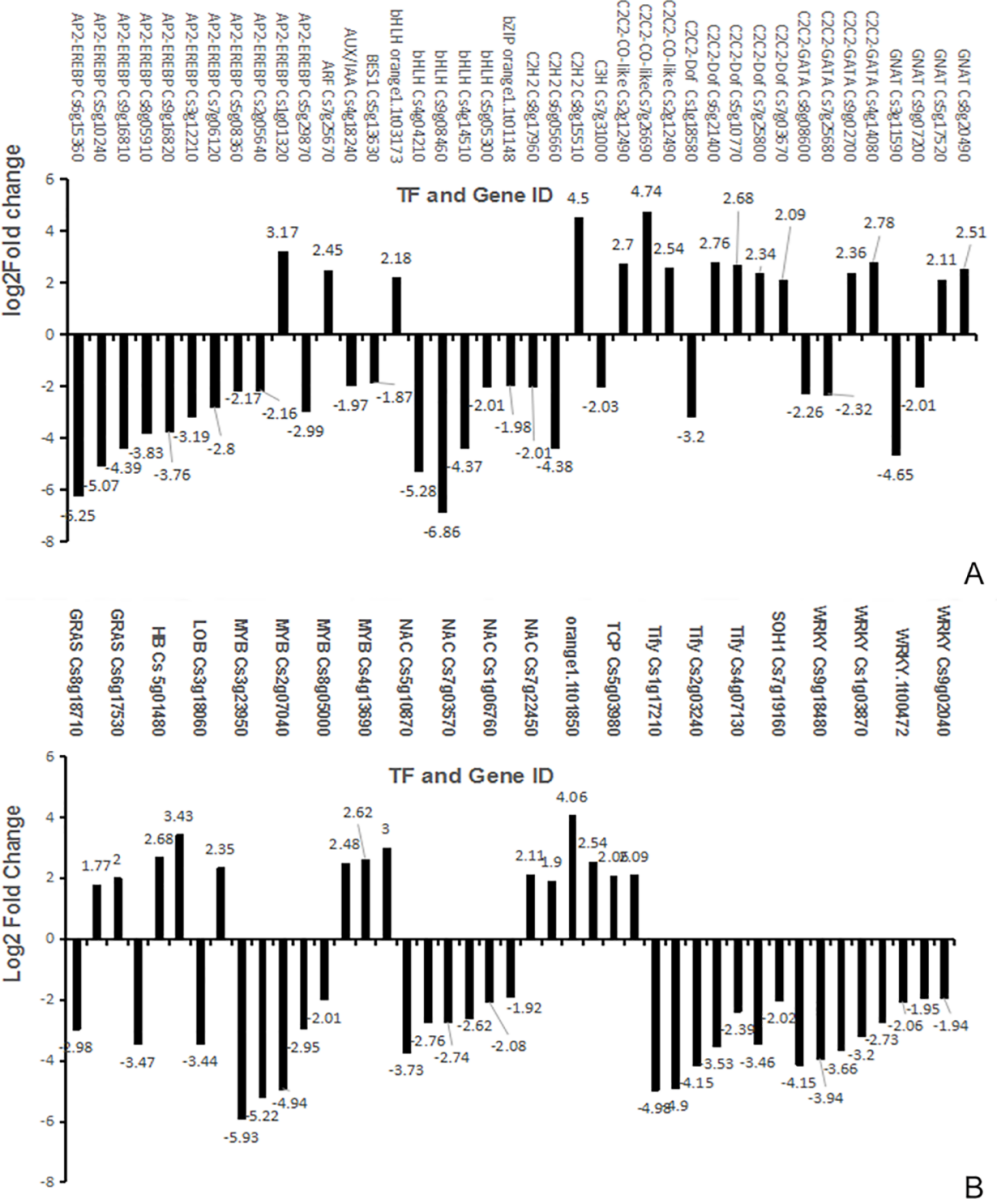
(A-B) Relative expression levels of TFs after *Bacillus* GJ1 treatment.

Many families of transcription factors interact in important ways with plant anti-adversity, hormone signaling, and trans-acting regulatory factors. Transcription factor analysis revealed changes in specific components after GJ1 treatment. Analysis of data from 135 transcriptomes revealed 83 differentially expressed TFs in the G66 versus the G11 groups; of these 83, 29 (21.5%) were upregulated, and 54 (40%) were downregulated in the G66 versus the G11 test group when the citrus plants were treated with *Bacillus* GJ1. Induction-responsive TFs belonging to the MYB, C2C2-Dof, C2C2-CO-like, C2C2-GATA, HB, C2C2-GATA, bHLH, LOB, Orphans, AP2-EREBP, NAC, ARF, TCP, GNAT, and GRAS families were upregulated (Fig 5A and 5B). Furthermore, several TFs related to plant development, including AUX/IAA, AP2-EREBP, BES1, bHLH, bZIP, C2C2-GATA, C2C2-GATA, C3H, C2H2, GRAS, GNAT, LOB, MYB, NAC, SOH1, TAZ, TRAF, Tify, and WRKY were downregulated (S8 Table, Fig 6A and 6B).

**Fig 7:**
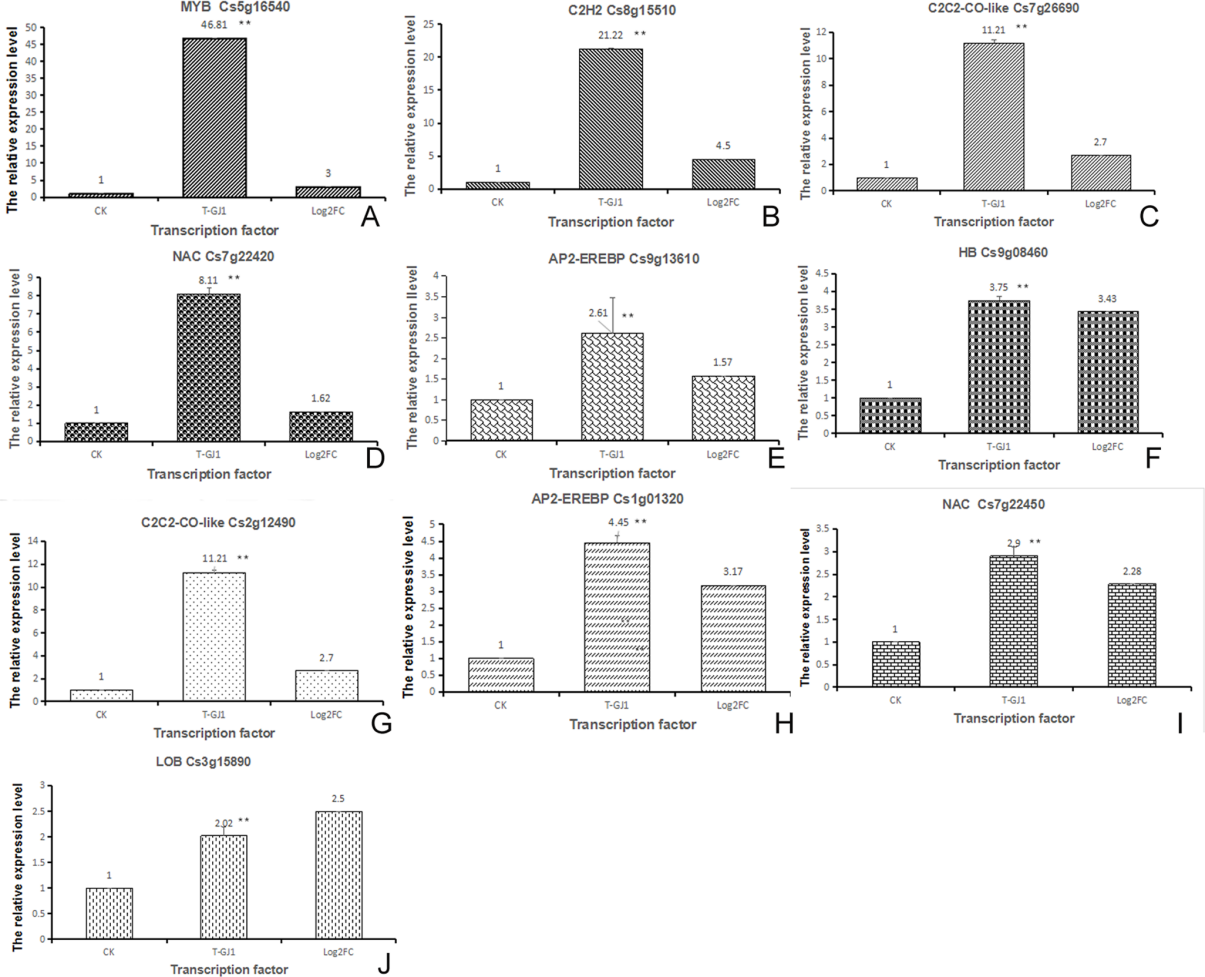
**(A-J) Validation of TFs by qPCR**. CK: control; T-GJ1: citrus treated with *Bacillus* GJ1; log_2_FC: results acquired from transcriptome data; *: significant difference (P < 0.05); **: extremely significant difference (P < 0.01).

To elucidate the correspondence between the mRNA transcript level and the log_2_-fold change of the individual transcripts in the transcriptome, we performed transcriptional analysis of 10 transcription factors (TFs) via qRT-PCR. The log_2_-fold changes (padj<0.05) in the transcription levels of 10 genes, including Cs5g16540 (MYB), Cs8g15510 (C2H2), Cs7g26690 (C2C2-CO-like), Cs7g22420 (NAC), Cs9g08460 (HB), Cs9g13610 (P2-EREBP), Cs2g 12490 (C2C2-CO-like), AP2-EREBP (Cs1g01320), Cs7g22450 (NAC), and Cs3g15890 (LOB), displayed trends similar to those displayed by the corresponding TFs (Fig 7A–7J).

### Primary data analysis and protein identification

**Fig 8:**
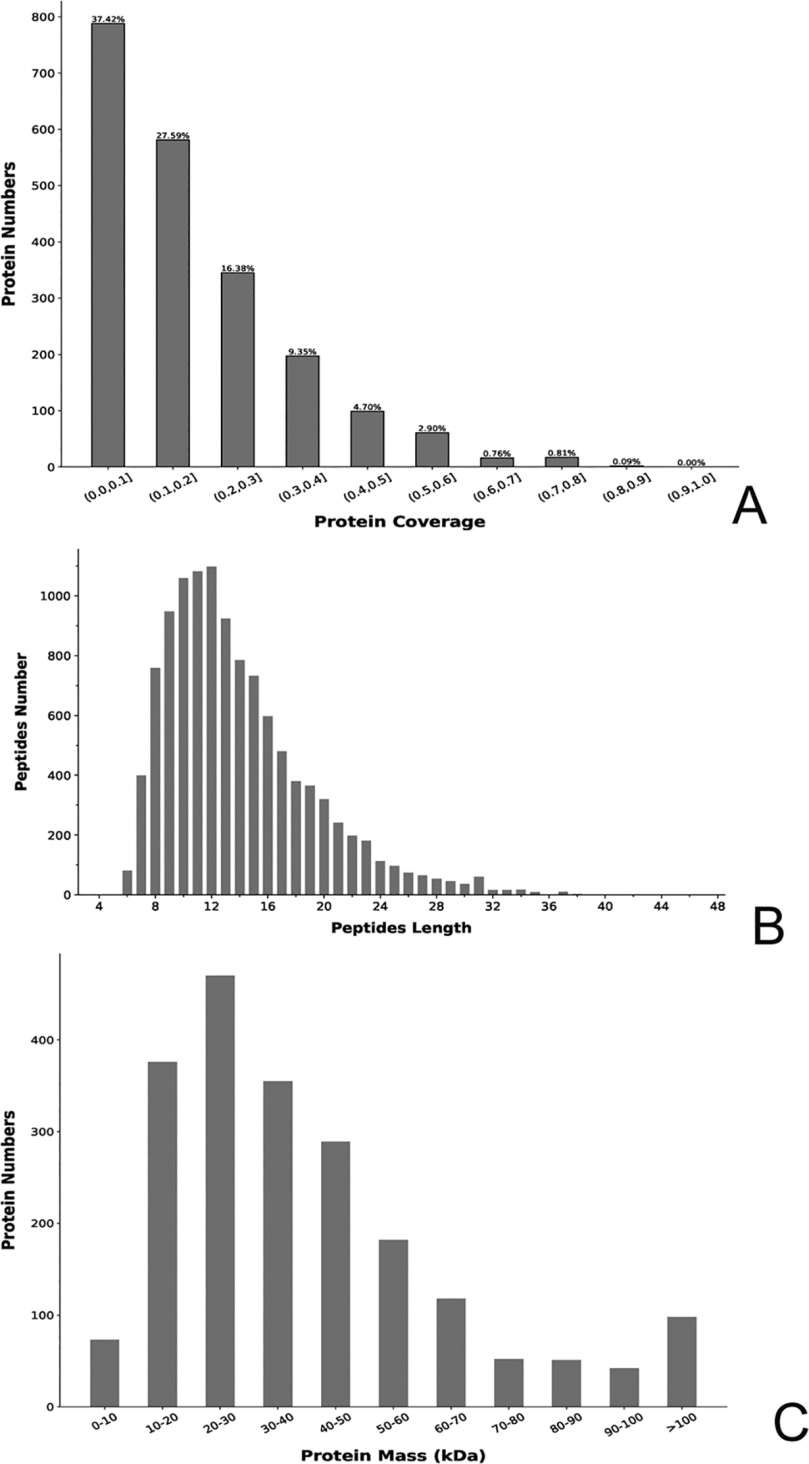
(A-C) Sequence coverage (A), distribution of length and number of peptides (B), and mass of proteins (C) identified by ITRAQ proteomics.

For protein identification, csipeptide.fasta (44,275 sequences) was used. A total of 326,502 spectra, 12,430 peptides, and 2779 proteins were generated from the ITRAQ experiment using the control and GJ1-treated leaves as the materials. From 9872 peptides, 8272 unique peptides were obtained (S9 Table). The mass and sequence coverage of proteins, the distribution of peptide length and the number of peptides, and the repeatability of the replicates are shown in Fig 8A–8C and S9 Table. Protein sequence coverage of 40–100%, 30–40%, 20–30%, 10–20%, and less than 10% accounted for 9.3%, 9.4%, 16.4%, 27.6%, and 37.4%, respectively, of the identified proteins. The 38 lowest-molecular-weight proteins (4.2 kDa) and the 1829 highest-molecular-weight proteins (217.4 kDa) were identified using the ITRAQ strategy.

### Gene Ontology (GO) functional classification analysis and genome (KEGG) analysis of proteins induced by *Bacillus* GJ1

**Fig 9:**
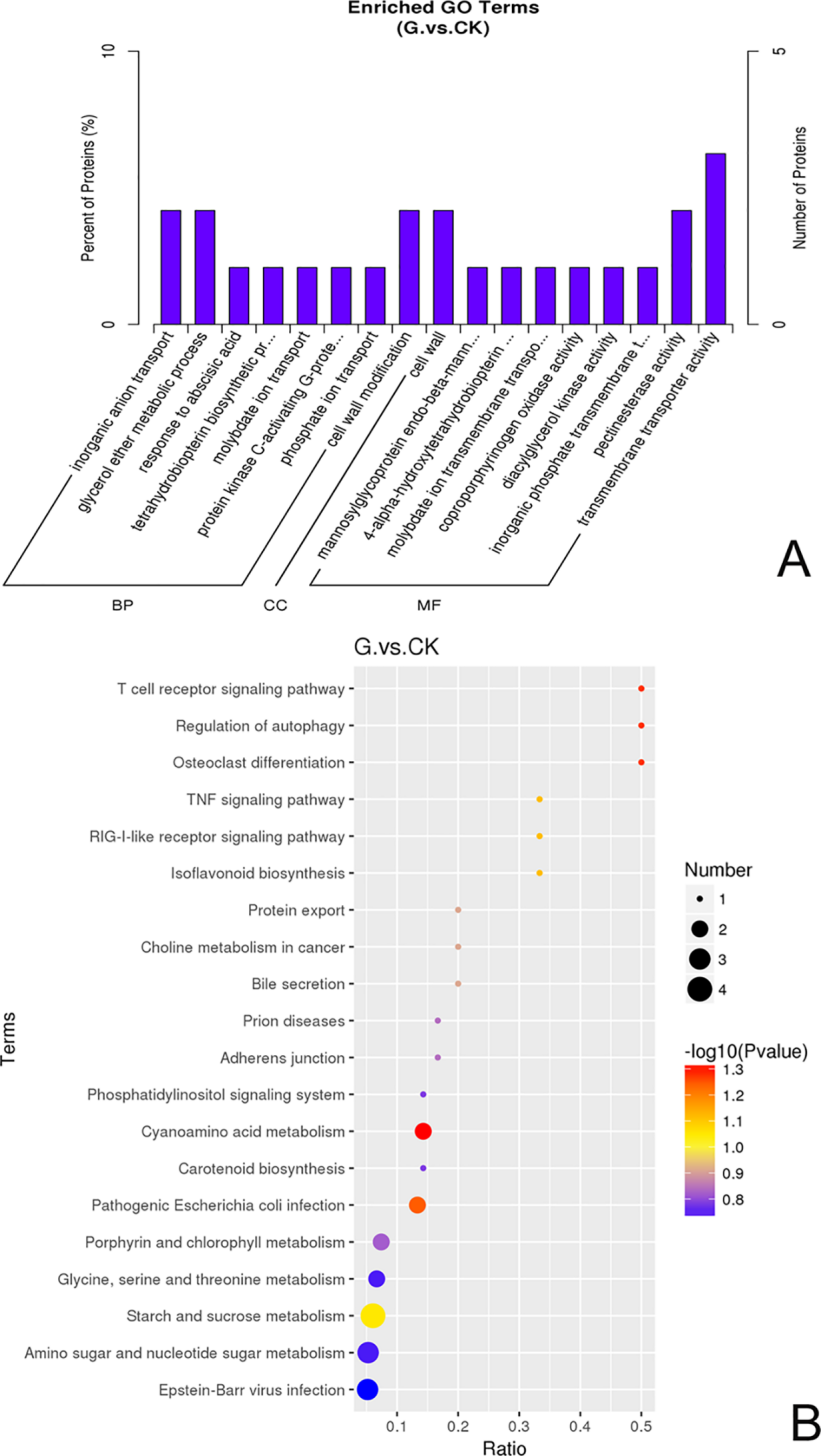
(A-B) Enriched GO terms (bar graph) and KEGG enrichment (scatter plot).

The protein expression levels in citrus plants treated with *Bacillus* GJ1 differed from those in the control plants. A total of 203 differentially expressed proteins were observed in *Bacillus*-treated citrus plants. We identified 52 enriched proteins that showed significant changes (padj < 0.05) in relative protein abundance in the treated citrus. Of these proteins, 20 were upregulated, and 32 were downregulated (S10 Table). According to the internationally standardized functional annotation classification, these proteins were primarily involved in biological processes, molecular functions, and cellular components (Fig 9A). The differential expression of the proteins was related to hydrolase activity (28.8%), ion transporter activity (25%), cell wall modification (13.5%), ATPase activity (3.8%), disulfide oxidoreductase activity (3.8%), cofactors (3.8%), chlorophyll synthesis (3.8%), kinase activity (3.8%), and other functions (11.4%).

Seventy-nine proteins were mapped to 60 pathways in the KEGG database to further investigate the biological functions of these proteins; a total of 2584 differentially expressed proteins were associated with these pathways. Cyanoamino acid metabolism was the most highly represented pathway, followed by autophagy regulation; isoflavonoid biosynthesis; starch and sucrose metabolism; protein export; porphyrin and chlorophyll metabolism; carotenoid biosynthesis; phosphatidylinositol signaling system; glycine, serine and threonine metabolism; and amino sugar and nucleotide sugar metabolism (padj < 0.578) (Fig 9B, S11 Table).

### Comparison of enriched KEGG pathways and GO with correlation analysis of the proteome and transcriptome

**Fig 10:**
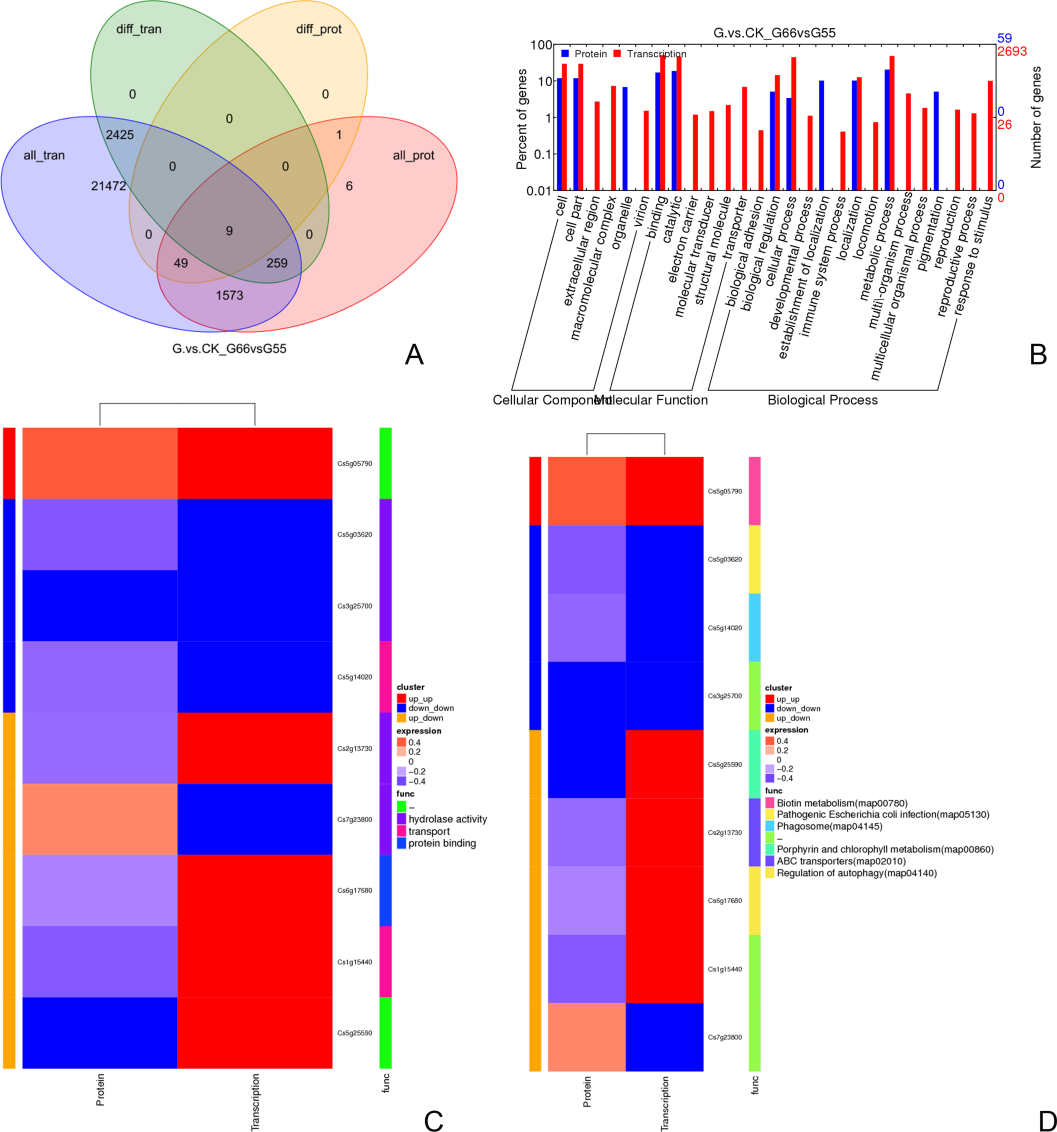
**(A-D) Correlation analysis of proteome and transcriptome**. A. Nine common and specific genes (proteins) obtained via corresponding relations are shown in a Venn diagram. In Fig 10A, all tran-all genes were obtained from the transcriptome; diff_tran-difference expression genes were identified by transcriptome analysis; Allprot-all proteins were identified by proteome analysis; diff_prot-difference proteins were identified by proteome analysis. B. Significantly differently expressed genes and significantly differently expressed proteins listed according to GO annotations. C and D. The common entries of the proteins that are significantly differently expressed and the genes that are significantly differently expressed in GO and KEGG, separately.

The mRNA information obtained from the transcriptome was integrated with the protein information identified by the proteome, and the corresponding relation is shown in a Venn diagram. Nine common and specific genes (proteins) were obtained by correlation analysis between differently expressed genes and different proteins, (Fig 10A, S12 Table). The genes (proteins) were identified conjointly by transcriptome and proteome, and multiple differences (log2 value) were analyzed by the correlation between the two groups. Each point in the figure represents a protein, and the green dot represents a protein with significant difference. The blue dot represents a protein with no significant difference (S9 Fig), The significantly differently expressed genes and the significantly differently expressed proteins are shown in the GO annotations list; these genes are involved in cells and cellular components; in binding and catalysis in the molecular function category; and in biological adhesion, cellular process, location, and metabolic process in the biological process category, and all of the genes were enriched in both ITRAQ and RNA-Seq data (Fig 10B). The common entries of the proteins that are significantly different in expression and of the genes that are significantly different in expression in GO, include hydrolase activity, transport, and protein binding (Fig 10C). The common entries of the proteins that are significantly different in expression and significantly different in expression in the KEGG pathway included proteins associated with phagosomes, regulation of autophagy, ABC transporters, pathogenic *Escherichia coli* infection, porphyrin and chlorophyll metabolism, and biotin metabolism (Fig 10D).

## Discussion

HLB severely hampers citrus production. The continuous increase of the incidence and spread of HLB is currently causing huge economic losses in citrus-producing countries. In this study, a potential biocontrol agent that acts against HLB pathogens, *B*. *amyloliquefaciens* GJ1, was identified, and the detoxification responses involved in its action were studied using transcriptome analysis, ITRAQ proteome analysis, and validation testing.

### *Bacillus amyloliquefaciens* GJ1 was screened and identified as a novel HLB antagonist

To mitigate and eliminate the adverse effects of HLB, a dynamic and sustainable method of biocontrol is desirable. This study successfully identified a biocontrol agent GJ1 that was effective to a certain extent against HLB, and sequence comparison and microtechniques identified GJ1 as a novel strain of *B*. *amyloliquefaciens*. The results showed that both the size and the shape of *Bacillus* GJ1 were consistent with *B*. *amyloliquefaciens* [24]. Resistance to HLB was evaluated using qPCR and PCR to detect the pathogen. The pathogen elimination rate of *Ca*. L. asiaticus reached 50% after irrigation of the infected plants seven times with an OD_600nm_≈1 *Bacillus* GJ1 solution. Bacillus GJ1 was newly registered in the China Center for Type Culture Collection (CCTCC).

In our experiments, a polypeptide was added to the plants that received *Bacillus* GJ1 and to the control plants; this polypeptide might have contributed to the disease resistance via interaction with GJ1. Previous studies have shown that some polypeptides can stimulate disease resistance in plants [39]. Pamela Ronald and other researchers developed a synthetic polypeptide that stimulates the XA21 pathway with exposure to a plant that expresses XA21, thereby increasing disease resistance in the plant [40]. In our experiments, the polypeptide also had an important synergistic effect (data not shown), and the *C*las-infected plants grew taller and stronger, and their root systems were well developed after treatment with both *Bacillus* GJ1 and the polypeptide.

### RNA-seq analysis revealed some of the pathways associated with the detoxification mechanism

#### Four significantly upregulated KEGG-enriched pathways

The physiological mechanisms of HLB disease are poorly understood because no genes encoding toxins, extracellular degrading enzymes, or specialized secretion systems have been found in the *Ca*. L. asiaticus genome. HLB causes starch accumulation in leaves [41], as shown by transcriptomic studies. This accumulation may block the transport of photosynthetic products and lead to yellowing and mottling of the leaves [19, 42]. A pathogen-induced host source-sink metabolic imbalance is likely the primary cause of the disease symptoms [12]. Although limited research on the mechanism of HLB disease resistance has been conducted, a few useful references are available. PRS2 is a resistance gene against *Pseudomonas syringae* bacteria that is induced by avirulence [43]. Mizrahi et al. (2009) [44] showed that the chaperone protein plays an important role in the degradation of misfolded proteins. In several citrus species (sweet orange, grapefruit, and lemon), the components of photosystems I and II are downregulated at the transcriptional and protein levels in Liberibacter-infected citrus [21, 45]. Notably, our RNA-seq analysis indicated that *Bacillus* GJ1 affected four significantly upregulated KEGG-enriched pathways (padj < 0.05): photosynthesis-antenna proteins, photosynthesis, protein processing in the endoplasmic reticulum, and plant-pathogen interactions. Genes with different relative expression levels included those involved in efficient light capture, thereby increasing photosynthetic capacity and improving the balance of carbohydrate metabolism and protein processing in the endoplasmic reticulum. We observed that 13 genes whose transcription is controlled by RPS2 were upregulated by *Bacillus* GJ1 treatment, and RPS2 further upregulates Hsp70 and HSP90, supporting the understanding that the expression of heat shock proteins is related to the hypersensitive response (HR) [46]. CDPK, CMCML, and MEKK1 were also upregulated, thereby increasing the defense-related responses of the plant. Additionally, increases in the energy metabolism, phenylalanine metabolism, and multiple secondary metabolite secretion were implicated in the protection of plants against *Ca*. L. asiaticus. Identification of those key upregulated pathways helps shed light on the possible regulation function of the photosynthesis and detoxification mechanisms involved in protection by *Bacillus* GJ1.

#### Upregulated KEGG-enriched pathways associated with secondary metabolites

The antagonistic activity of biocontrol agents such as antibiotics and pyoverdine siderophores against phytopathogenic fungi may have different mechanisms [47]. Secondary metabolite secretion is the most important mechanism through which biocontrol agents suppress pathogens. Bacilli can produce many types of antibiotics, including polypeptide antibiotics [48, 49], lipopeptides [50], polyketides [51, 52], amino acids, and volatile compounds [50]. Wu et al. (2015) [53] reported that FZB42 produces antibiotics and causes bacilysin overproduction in *B*. *amyloliquefaciens* and that FZB42 and FZBSPA increase antibacterial activity, stimulate plant growth, and suppress plant pathogens. We also observed upregulation of the production of multiple secondary metabolites after Bacillus GJ1 treatment; these metabolites included stilbenoids, diarylheptanoids, gingerols, flavonoids, isoquinoline alkaloids, steroids, tropane, piperidine, pyridine alkaloids, compounds with terpenoid backbones, and phenylpropanoids. In the upregulated, KEGG-enriched pathways (padj < 0.05), these secondary metabolites could suppress competitive bacteria and play an important role in disease resistance.

### Proteome analysis revealed some of the pathways associated with the detoxification mechanism

The proteomic data obtained in this study indicated that the upregulated expression of specific proteins might play a role in plant resistance to bacterial diseases. Cao et al. (2013) [32] suggested that energy metabolism, biosynthesis, and stress resistance have important roles in the antagonism exhibited by strain G1. Zhang et al. [54] demonstrated that *Y*. *lipolytica* stimulates the activities of polyphenol oxidase, peroxidase, chitinase, and L-phenylalanine ammonia lyase, which are involved in increasing the defense responses in apple. Cyanoamino acid is a neurotoxic non-protein amino acid found in the seeds of wild peas; its production is related to stress [55]. The autophagic degradation of cytoplasmic components regulates age- and immunity-related programmed cell death [56]. The serine/threonine kinase gene is a key gene associated with powdery mildew resistance [57]. In this study, cyanoamino acid metabolism; autophagy regulation; glycine, serine, and threonine metabolism; isoflavonoid biosynthesis; starch and sucrose metabolism; porphyrin and chlorophyll metabolism; carotenoid biosynthesis; and amino sugar and nucleotide sugar metabolism were upregulated in terms of KEGG-enriched proteins. The upregulation of these pathways might form the basis for the observed resistance to HLB. Validation of the increased expression of these important proteins was performed in our study.

### TFs could be important components of the *Bacillus* GJ1-induced defense response and function regulation

TFs regulate genes to ensure their expression in the correct cells at the correct time and in the correct amount throughout the life cycle of cells and organisms. In plants, the most common TFs involved in disease resistance are AP2/ERF (ethylene response factor), MYB (γ-myb avian myeloblastosis viral oncogene homolog), bZIP (basic leucine-zipper), WRKY (N-containing WRKYGQK high conservative sequence) [58], NAC (NAM-ATAFI1/2 and CUC2) [59], and others. The TFs AP2/ERF are primarily involved in jasmonic acid (JA)- and ethylene (ET)-mediated signal transduction, whereas WRKY and bZIP TFs are primarily involved in salicylic acid (SA)-mediated signal transduction. In combination with other transcription factors, AP2/ERF proteins may regulate crosstalk and the expression of various genes involved in plant defenses [60, 61]. Previous reports indicate that MYB68 expression is upregulated in Las-infected citrus [61]. The overexpression of WRKY4 increases the susceptibility of *Arabidopsis* plants to *P*. *syringae* and suppresses PR1 gene expression, but the effect of upregulation of WRKY4 in Las-infected citrus remains to be addressed [62]. Citrus homologs of WRKY6 and WRKY40 are also upregulated at the transcriptional level in sweet oranges infected with Lam [63].

In this study, 29 TFs were upregulated and 54 were downregulated in G66 compared with those in the G11 group. The TFs apparently participated in the regulation of various functions in our tests of Las-infected citrus treated and untreated with GJ1. We analyzed some of the functions of the upregulated TFs. The ERF/AP2 domain is involved in dehydration and cold-inducible gene expression in *Arabidopsis* [64]. Citrus homologs of ERF/AP2 (Cs1g01320) and ERF (Cs9g13610) were upregulated at the transcriptional level in Las-infected plants treated with GJ1. Orphans (Cs2g24030), C2C2-CO-like (Cs7g26690), and C2C2-CO-like (Cs2g12490) and the homologous gene of the chloroplast import apparatus (CIA2) were all upregulated after GJ1 treatment. The ascorbate oxidase promoter-binding protein (AOBP) is highly conserved in many plant proteins and is significantly related to steroid hormone receptors [65]. Our study indicated that the homologs C2C2-Dof (Cs7g03670) and C2C2-Dof (Cs5g10770) were upregulated after GJ1 treatment. bHLH122 is important for drought and osmotic stress resistance in *Arabidopsis* and in the repression of ABA catabolism [66]. In this study, bHLH (orange1.1t03173) was upregulated after GJ1 treatment. Auxin response factors (ARFs) bind specifically to the DNA sequence 5'-TGTCTC-3' found in auxin-responsive promoter elements [67]. In our tests, ARF (Cs7g25670) was upregulated after GJ1 treatment. The NAC100 binds to the promoter regions of genes involved in chlorophyll catabolism [68], and NAC (Cs7g22450) and NAC (Cs7g22420) were upregulated after GJ1 treatment. Additionally, MYB (Cs5g16540) may participate in phytochrome regulation, and C2H2 (Cs8g15510) may have DNA-binding activity. The Cs5g08460 encodes a homeobox-leucine zipper protein in *Glycine max* that may act as a mediator of red/far-red light effects on leaf cell expansion in the shading response. LOB domain-containing protein 39 (Cs3g15890) may regulate later organ boundaries (S8 Table). These functions of TFs are also reported in other organisms [69–72]. We verified 10 upregulated TFs via qPCR. TFs are potentially important factors in the defense response and may be important in the elimination of the HLB pathogen and in the regulation of metabolic balance. The regulation of the expression of specific TFs observed in this study provided valuable clues regarding the mechanism of control of HLB disease by Bacillus GJ1.

### Comprehensive understanding of the detoxification responses based on the transcriptomes and proteomes

**Fig 11:**
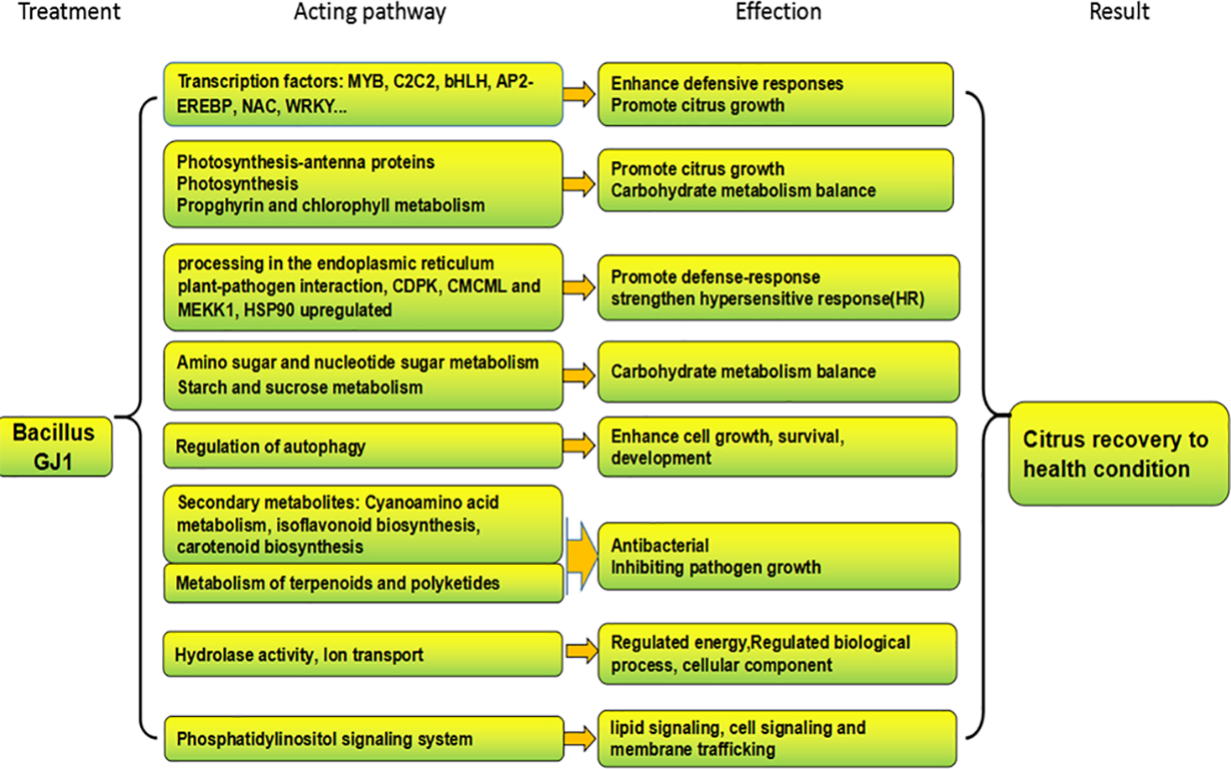
Hypothetical detoxification mechanism for the elimination of the HLB pathogen by GJ1 treatment.

The results of this study, which employed both transcriptome and proteome analyses, strongly suggest that the mechanism of HLB pathogen elimination by GJ1 was based on multiple factors rather than on a single defensive reaction. Our results demonstrated that GJ1 induces TF regulation of multiple genes involved in important metabolic pathways and that this regulation may facilitate several defensive responses, including increased light capture capacity, light absorption, photosynthesis, and growth; upregulation of heat shock proteins to force chaperones and folding catalysts; degradation of misfolded proteins; strengthening of the hypersensitive response (HR); increased production of multiple secondary metabolites to inhibit pathogen growth; promotion of the digestion of superfluous metabolic products and maintenance of metabolic balance; strengthening of energy metabolism through ion transport activity; and regulation of autophagy for degrading damaged cells and metabolic waste (Fig 10). Therefore, the HLB-infected citrus plants can recover to a condition of health after GJ1 treatment according to the hypothesis shown in Fig 11.

### Strategies for the biocontrol of HLB in citrus production

Although the in vitro selection of bacteria antagonistic to uncultured *Ca*. L. asiaticus is difficult, we successfully screened and tested antagonistic bacteria using potted plants. This process has been shown to be an effective method for rapidly obtaining available antagonistic bacteria. Once detoxification strains appear, the antagonistic bacteria can be evaluated quickly and used for protection against HLB in vivo. This screening strategy for biocontrol agents can also be applied to similar uncultivated pathogens; thus, this study provides a usable model for such isolation. However, much work remains, such as the detection of *Ca*. L. asiaticus with a hypersensitive Taqman-based PCR method, screening for the optimal biologic titer and dosage, distinguishing live vs. dead cells of *C*Las with dye treatment and PCR detection, and observing the stability and persistence of the biocontrol by GJ1, among others.

Our study indicated that the rate of detoxification reached 50% after potted plants were irrigated continuously seven times with an OD_600nm_≈1 *Bacillus* GJ1 solution. Additional experiments in which the concentration of the bacterial suspension and the duration of the treatment varied are required to maximize the detoxification rate for its application in the biocontrol of HLB. Combining antagonism and shoot tip grafting offers potential for use in virus-free citrus seedling propagation and in the development of new varieties of healthy nursery stock. This biocontrol process is also beneficial for modern net house planting of high-quality citrus varieties.

## Conclusions

Bacillus GJ1 was screened and identified as *B*. *amyloliquefaciens*. The rate of detoxification reached 50% after seven treatments of potted *C*las-infected plants with an OD_600nm_≈1 GJ1 solution. Transcriptome and ITRAQ proteome analyses revealed that several important pathways were upregulated by the *B*. *amyloliquefaciens* GJ1. This study highlights novel strategies that can be used for HLB biocontrol in nursery-grown citrus plants and to improve the detoxification efficiency.

## Supporting information

S1 Fig Phylogenetic tree alignment of 16SrDNA and amplification standard curve of *rplJ and COX*. (TIF)

S2 Fig Percentage of mapped gene orgion. (TIF)

S3 Fig Photosynyesis-antenna protein. (TIF)

S4 Fig Photosynthesis. (TIF)

S5 Fig Protein processing in endoplasmic reticulum. (TIF)

S6 Fig Plant-pathogen interaction. (TIF)

S7 Fig Porphyrin and chlorophyll metabolism. (TIF)

S8 Fig Amplification curve and melting curve. (TIF)

S9 Fig Correlation of the genes (proteins) in transcriptome and proteome. (TIF)

S1 Table Primers sequence and data for Fig 2. (XLSX)

S2 Table Data output quality and read distribution. (XLSX)

S3 Table DEG GO enrichment up and down. (XLSX)

S4 Table G66 versus G55, G55 versus G11 KEGG up. (XLSX)

S5 Table G66 versus G55, G55 versus G11 KEGG down. (XLSX)

S6 Table G66 versus G11 KEGG down. (XLSX)

S7 Table Gene ID for heatmap. (XLS)

S8 Table Comparison information of transcript factors. (XLSX)

S9 Table Proteome primary data. (XLSX)

S10 Table GJ1 versus CK GO Enrich merge updown. (XLSX)

S11 Table GJ1 versus CK KEGG Enrich. (XLS)

S12 Table Correlation analysis of proteome and transcripteom. (XLSX)

## Abbreviations

BCA: Biocontrol agent
BLAST: Bell labs layered space-time
ERAD: ER-associated degradation
*C*Las: *Candidatus* Liberibacter asiaticus
GO: Gene ontology
FPKM: Expected number of fragments per kilobase of transcript sequence per millions base pairs sequenced
HLB: Huanglongbing
KEGG: Kyoto Encyclopedia of Genes and Genomes
NCBI: National Center for Biotechnology Information
PAMP: Pathogen-associated molecular pattern
qPCR: Real-time quantitative PCR detecting system
TEM: Transmission electron microscopy
ITRAQ: Tags for relative and absolute quantification of the proteome
