# Supplementary figures and images for: Transcriptome sequencing and ITRAQ reveal the detoxification mechanism of Bacillus GJ1, a potential biocontrol agent for Huanglongbing
Source: PLoS One. 2018 Aug 9;13(8):e0200427. doi: 10.1371/journal.pone.0200427 (PMC6084860; doi:10.1371/journal.pone.0200427)

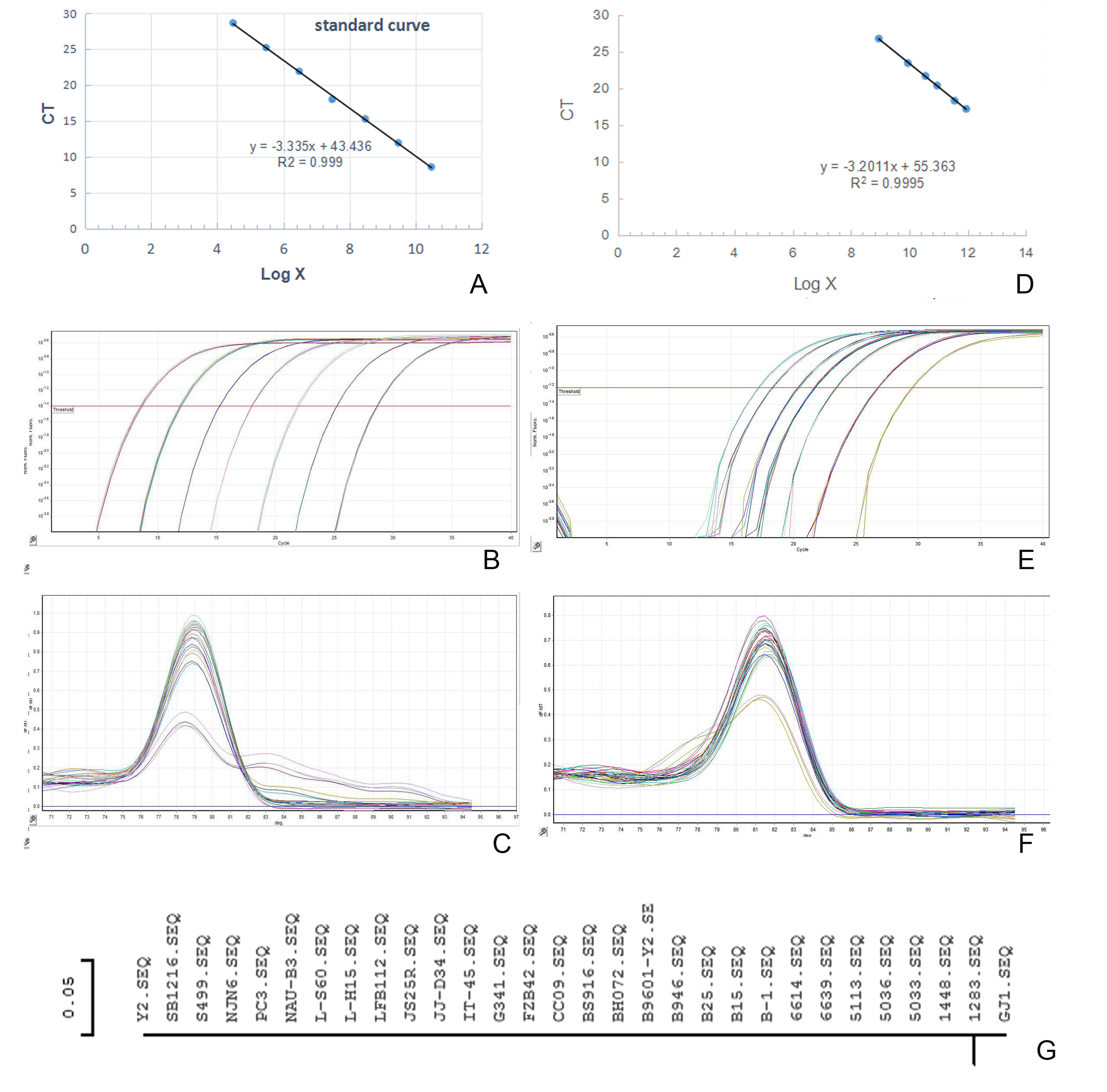

Supplement: S1 Fig — (TIF) [file pone.0200427.s001.tif]

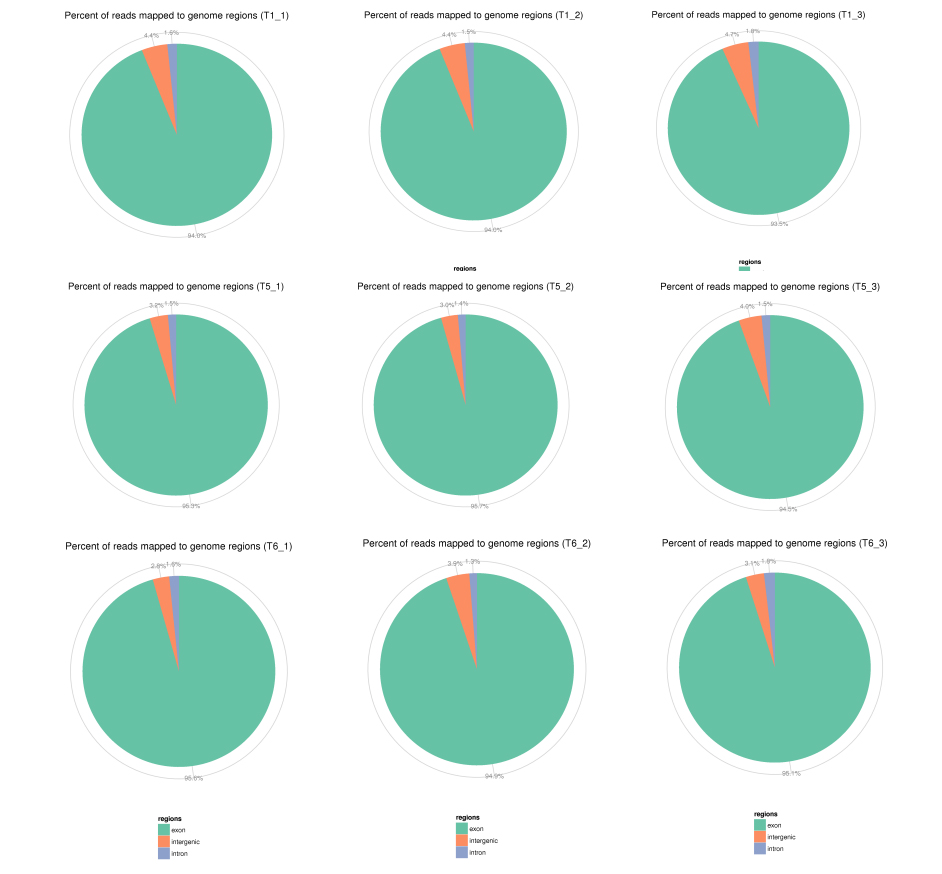

Supplement: S2 Fig — (TIF) [file pone.0200427.s002.tif]

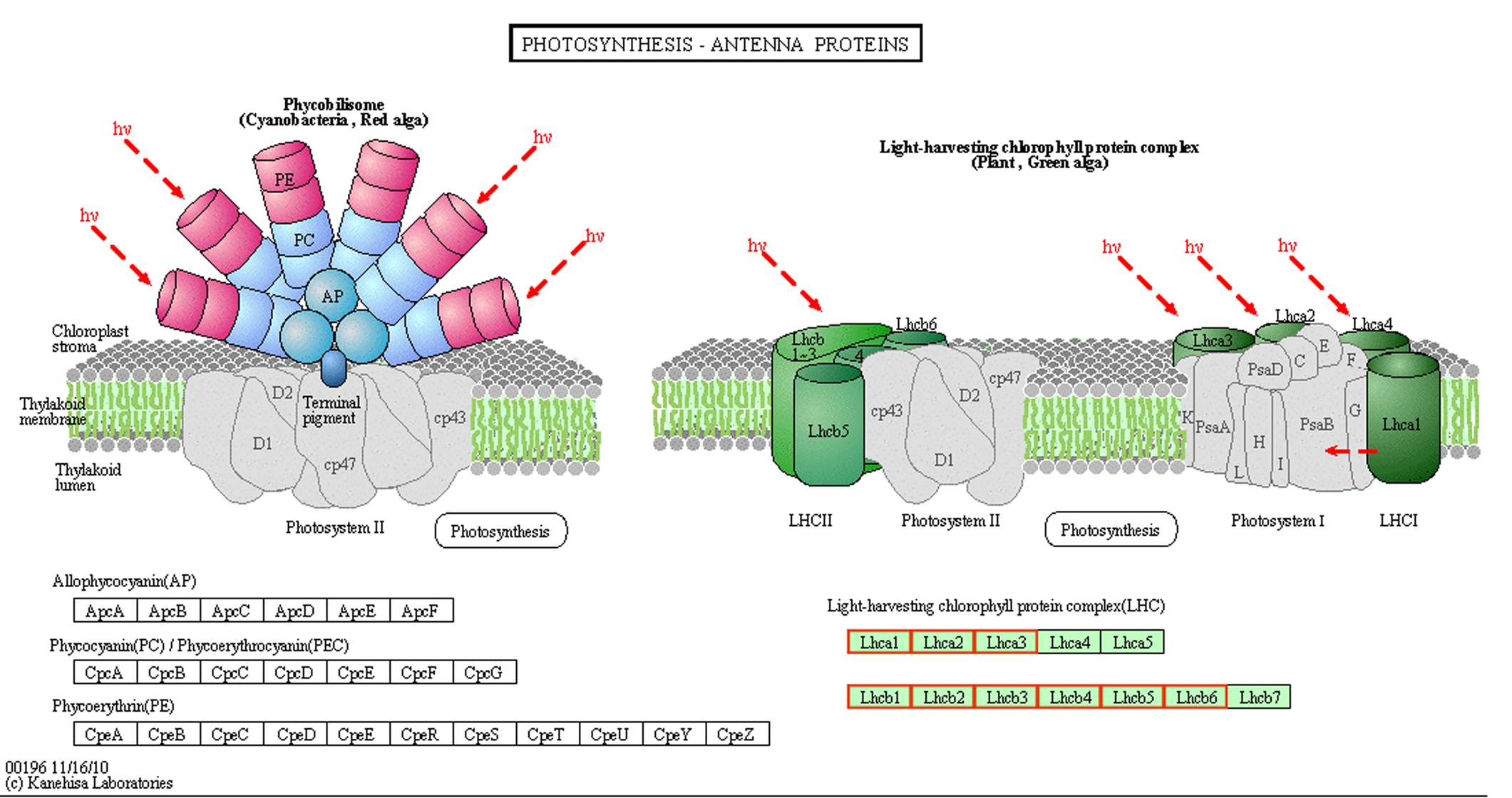

Supplement: S3 Fig — (TIF) [file pone.0200427.s003.tif]

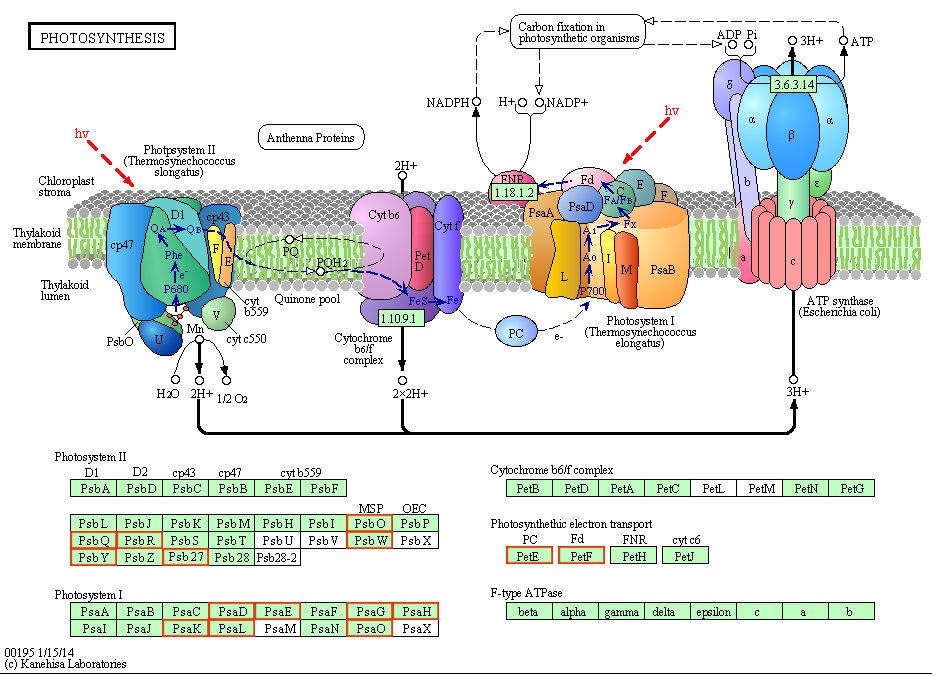

Supplement: S4 Fig — (TIF) [file pone.0200427.s004.tif]

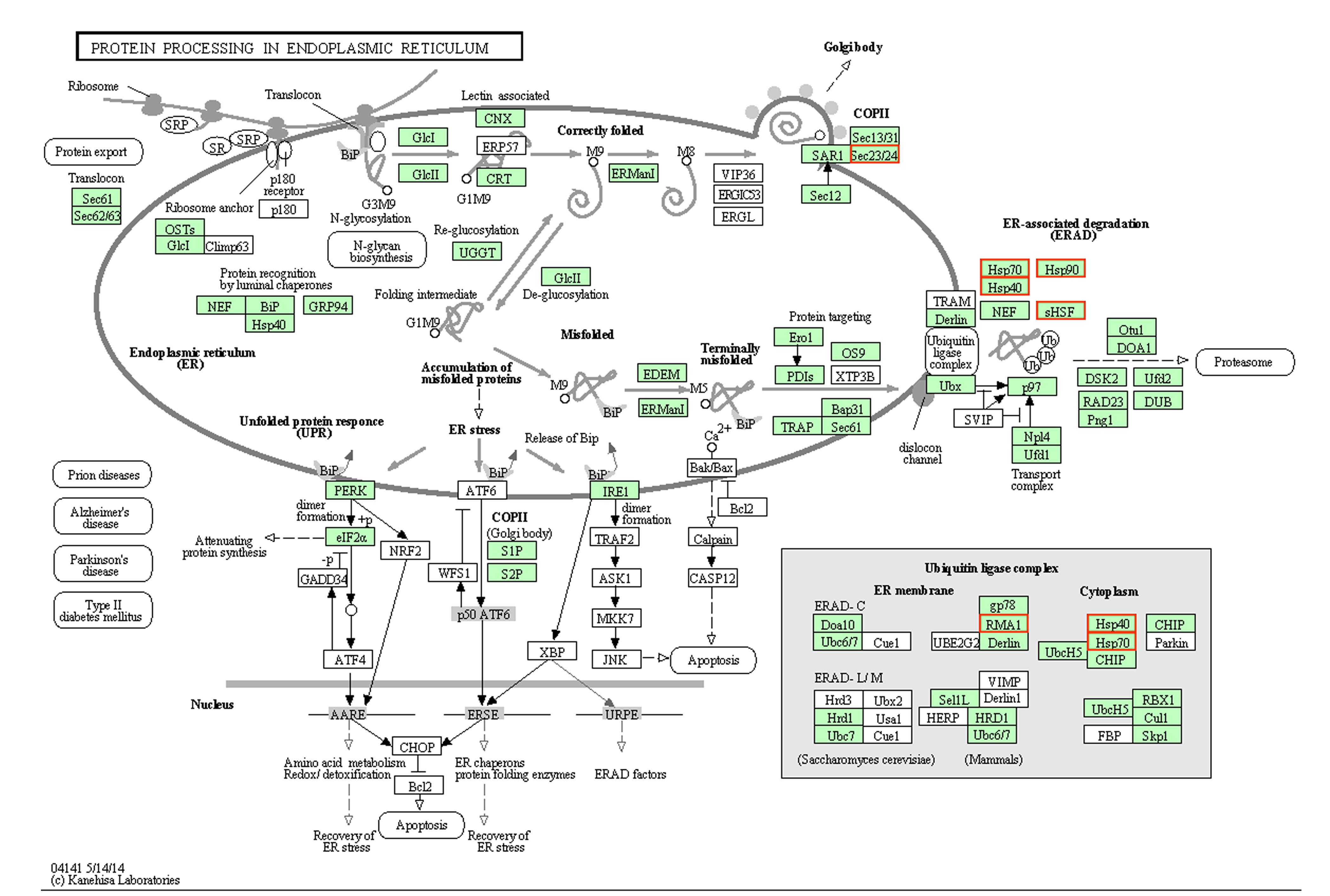

Supplement: S5 Fig — (TIF) [file pone.0200427.s005.tif]

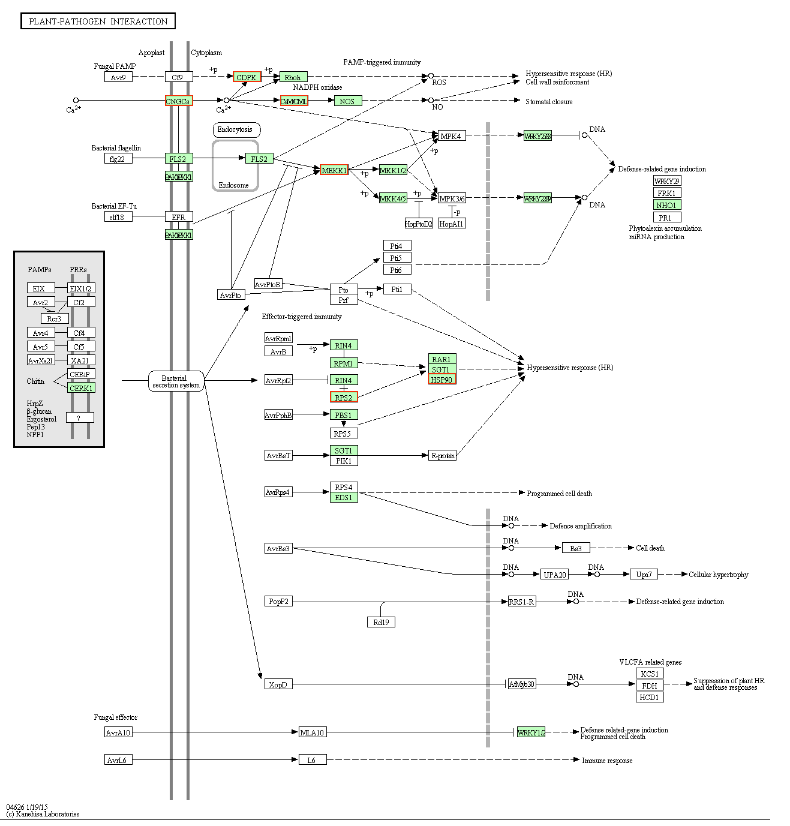

Supplement: S6 Fig — (TIF) [file pone.0200427.s006.tif]

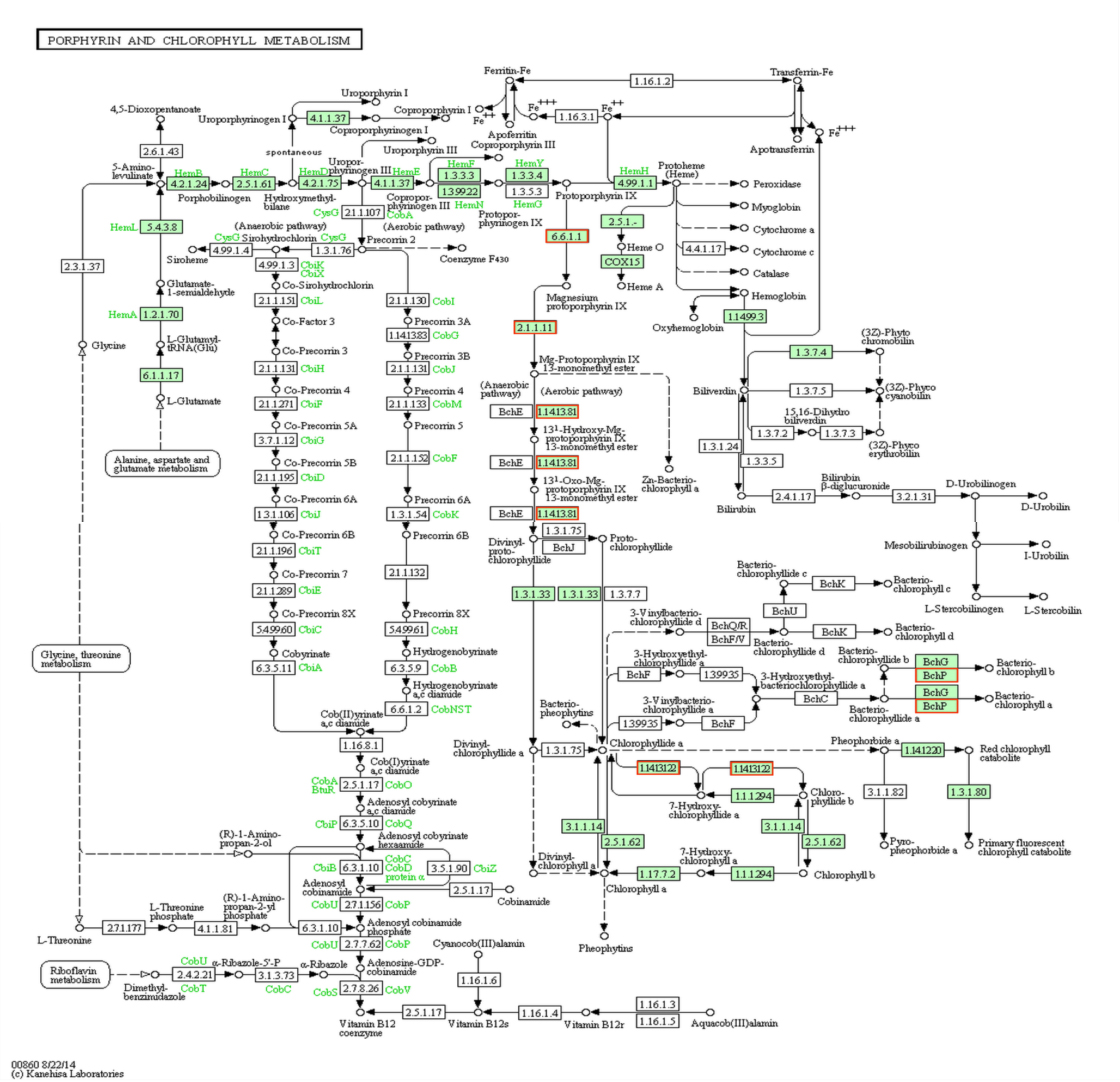

Supplement: S7 Fig — (TIF) [file pone.0200427.s007.tif]

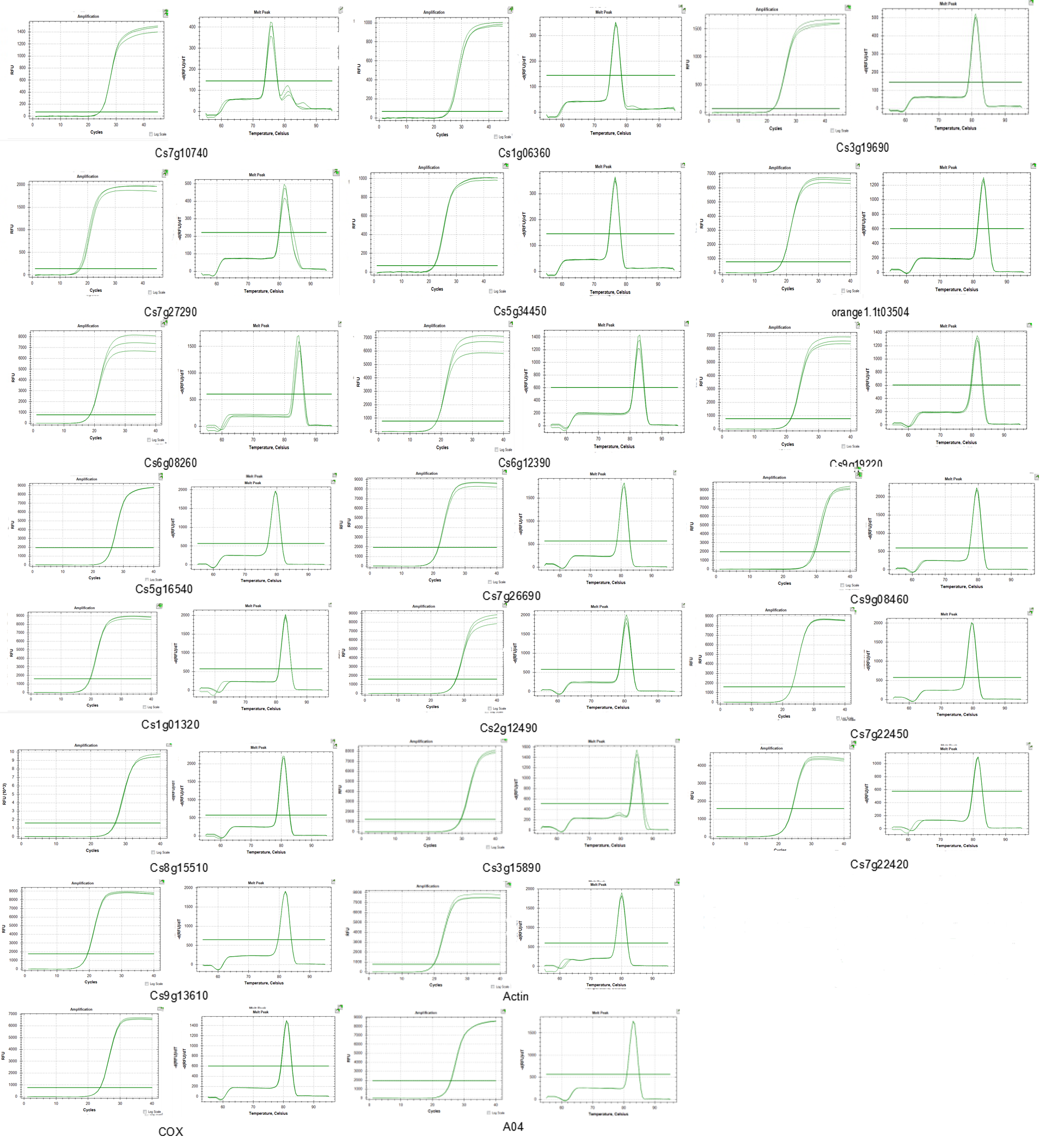

Supplement: S8 Fig — (TIF) [file pone.0200427.s008.tif]

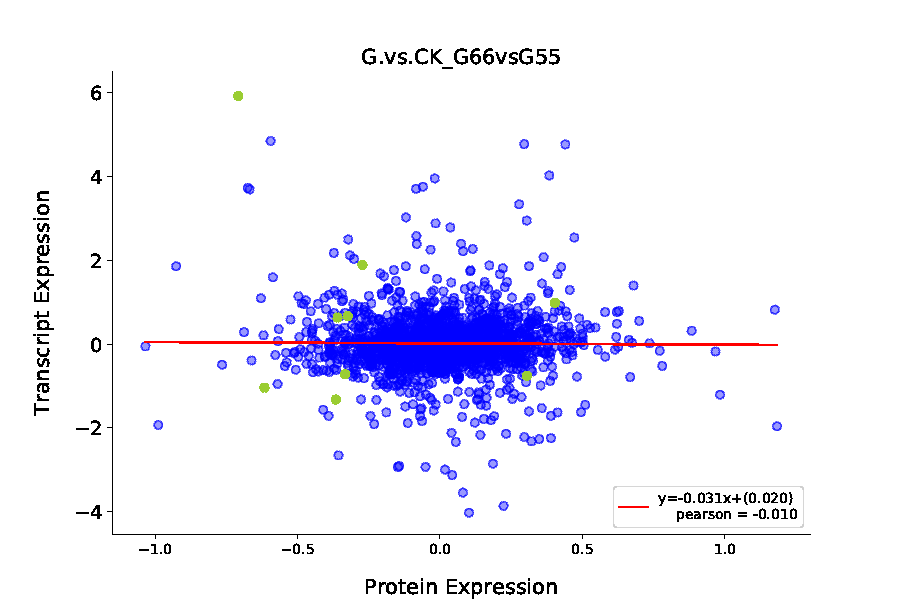

Supplement: S9 Fig — (TIF) [file pone.0200427.s009.tif]
